# Long-term exposure to transportation noise and obesity: A pooled analysis of eleven Nordic cohorts

**DOI:** 10.1097/EE9.0000000000000319

**Published:** 2024-07-08

**Authors:** Åsa Persson, Andrei Pyko, Lara Stucki, Mikael Ögren, Agneta Åkesson, Anna Oudin, Anne Tjønneland, Annika Rosengren, David Segersson, Debora Rizzuto, Emilie Helte, Eva M. Andersson, Gunn Marit Aasvang, Hrafnhildur Gudjonsdottir, Jenny Selander, Jesper H. Christensen, Karin Leander, Kristoffer Mattisson, Kristina Eneroth, Lars Barregard, Leo Stockfelt, Maria Albin, Mette K. Simonsen, Mårten Spanne, Nina Roswall, Pekka Tiittanen, Peter Molnár, Petter L.S. Ljungman, Satu Männistö, Tarja Yli-Tuomi, Thomas Cole-Hunter, Timo Lanki, Youn-Hee Lim, Zorana J. Andersen, Mette Sørensen, Göran Pershagen, Charlotta Eriksson

**Affiliations:** a Institute of Environmental Medicine, Karolinska Institutet, Stockholm, Sweden; b Center for Occupational and Environmental Medicine, Region Stockholm, Stockholm, Sweden; c Department of Occupational and Environmental Medicine, Sahlgrenska University Hospital, Gothenburg, Sweden; d Occupational and Environmental Medicine, School of Public Health and Community Medicine, Institute of Medicine, University of Gothenburg, Gothenburg, Sweden; e Occupational and Environmental Medicine, Lund University, Lund, Sweden; f Danish Cancer Institute, Strandboulevarden 49, 2100 Copenhagen Ø, Denmark; g Department of Public Health, University of Copenhagen, Copenhagen, Denmark; h Department of Molecular and Clinical Medicine, Institute of Medicine, Sahlgrenska Academy, University of Gothenburg, Gothenburg, Sweden; i Region Västra Götaland, Department of Medicine Geriatrics and Emergency Medicine, Sahlgrenska University Hospital Östra Hospital, Gothenburg, Sweden; j Swedish Meteorological and Hydrological Institute, Norrköping, Sweden; k Aging Research Center, Department of Neurobiology Care Science and Society, Karolinska Institutet and Stockholm University, Stockholm, Sweden; l Stockholm Gerontology Research Center, Stockholm, Sweden; m Department of Air Quality and Noise, Norwegian Institute of Public Health, Oslo, Norway; n Centre for Epidemiology and Community Medicine, Region Stockholm, Stockholm, Sweden; o Department of Global Public Health, Karolinska Institutet, Stockholm, Sweden; p Department of Environmental Science, Aarhus University, Roskilde, Denmark; q Division of Occupational and Environmental Medicine, Lund University, Lund, Sweden; r Environment and Health Administration, Stockholm, Sweden; s Department of Neurology and the Parker Institute, Frederiksberg Hospital, Frederiksberg, Denmark; t Environment Department, City of Malmö, Malmö, Sweden; u Department of Health Security, Finnish Institute for Health and Welfare, Kuopio, Finland; v Department of Cardiology, Danderyd Hospital, Stockholm, Sweden; w Department of Public Health and Welfare, Finnish Institute for Health and Welfare, Helsinki, Finland; x Institute of Public Health and Clinical Nutrition, University of Eastern Finland, Kuopio, Finland; y Department of Environmental and Biological Sciences, University of Eastern Finland, Kuopio, Finland; z Department of Natural Science and Environment, Roskilde University, Denmark

**Keywords:** Transportation noise, Obesity, Overweight, Central obesity, BMI, Waist circumference, Exposure-response association, Air pollution

## Abstract

**Background:**

Available evidence suggests a link between exposure to transportation noise and an increased risk of obesity. We aimed to assess exposure-response functions for long-term residential exposure to road traffic, railway and aircraft noise, and markers of obesity.

**Methods:**

Our cross-sectional study is based on pooled data from 11 Nordic cohorts, including up to 162,639 individuals with either measured (69.2%) or self-reported obesity data. Residential exposure to transportation noise was estimated as a time-weighted average L_den_ 5 years before recruitment. Adjusted linear and logistic regression models were fitted to assess beta coefficients and odds ratios (OR) with 95% confidence intervals (CI) for body mass index, overweight, and obesity, as well as for waist circumference and central obesity. Furthermore, natural splines were fitted to assess the shape of the exposure-response functions.

**Results:**

For road traffic noise, the OR for obesity was 1.06 (95% CI = 1.03, 1.08) and for central obesity 1.03 (95% CI = 1.01, 1.05) per 10 dB L_den_. Thresholds were observed at around 50–55 and 55–60 dB L_den_, respectively, above which there was an approximate 10% risk increase per 10 dB L_den_ increment for both outcomes. However, linear associations only occurred in participants with measured obesity markers and were strongly influenced by the largest cohort. Similar risk estimates as for road traffic noise were found for railway noise, with no clear thresholds. For aircraft noise, results were uncertain due to the low number of exposed participants.

**Conclusion:**

Our results support an association between road traffic and railway noise and obesity.

**What this study adds**

The results of this large-scale Nordic multi-center study add to the evidence of an association between long-term exposure to road traffic and railway noise and obesity, with suggested thresholds of 50–55 dB and 55–60 dB L_den_ for road traffic noise and obesity, and central obesity, respectively, and a 10% risk increase per 10 dB thereafter. Thus, these results suggest a potential pathway between transportation noise and the development of cardiometabolic diseases.

## Introduction

Environmental noise is increasingly recognized as a risk factor for cardiovascular and metabolic diseases, such as ischemic heart disease (IHD), stroke, and type 2 diabetes.^1^ Harmful levels of environmental noise are estimated to affect at least 20% of the EU population, cause 12,000 premature deaths, and contribute to 48,000 new cases of IHD per year in Europe.^2^ It is estimated that 22 million people are highly annoyed by transportation noise and 6.5 million are highly sleep disturbed. In view of the new evidence on the adverse effects of transportation noise and the significant public health implications, the World Health Organization (WHO) proposed stricter environmental noise guidelines for the European Region in 2018.^3^

Overweight and obesity are important risk factors for cardiovascular disease and type 2 diabetes and thus present a possible pathway between noise and cardiometabolic outcomes.^1^ Several studies have assessed the association between exposure to transportation noise and overweight and obesity. In 2022, the epidemiological evidence was summarized in a systematic review and meta-analysis of six cross-sectional and seven longitudinal studies, including nine from Nordic countries, one from Bulgaria, one from Slovakia, one from Switzerland, and one from the Netherlands.^4^ Associations were observed between exposure to each of the three transportation noise sources (i.e., road traffic, railway, and aircraft noise) and different measures of obesity, in particular with waist circumference (WC) and central obesity. For instance, Gui et al.^4^ found an increase in WC of 0.158 cm (95% confidence interval [CI] = 0.08, 0.24) per 10 dB day-evening-night level (L_den_) increment. Less conclusive results were, however, found for other obesity indicators such as body mass index (BMI) and waist-hip ratio. No attempt was made to assess the shape of exposure-response functions, although there is evidence of thresholds for other noise-induced health outcomes such as cardiovascular disease.^5^

Causal pathways for the effects of noise on adiposity markers may involve sleep disturbance and psychological stress.^1^ For instance, sleep deprivation may lead to dysregulation of appetite-regulating hormones such as leptin and ghrelin, which leads to an increased appetite and reduced energy expenditure, thus contributing to overweight and obesity.^6^ Noise-induced sleep disturbance has been observed to result in weight gain in experimental animals.^7^ Furthermore, noise may act as a stressor, activating the hypothalamic-pituitary axis and elevating the levels of stress hormones such as cortisol, thereby promoting central fat deposition.^8,9^ It has also been shown that participants living near airports have elevated saliva cortisol levels related to noise exposure.^10^

Combined exposure to several environmental factors, such as noise from different modes of transportation and air pollution, may be particularly harmful but has been investigated only to a limited extent in relation to adiposity markers.^11,12^ Additionally, to increase the understanding of etiological pathways and for effective prioritization of preventive measures, more evidence is needed regarding the effect modification of the association between transportation noise exposure and obesity markers by sociodemographic variables, for instance, sex, age, and education, as well as different lifestyle characteristics, including smoking and physical activity.

The aim of this study was to comprehensively assess exposure-response relationships between road traffic, railway and aircraft noise, and obesity markers, including both BMI and WC. Furthermore, we aimed to investigate potential effect modification by air pollution, sociodemographic characteristics, and lifestyle factors.

## Methods

### Study population

This study is a pooled analysis of 11 Nordic cohorts in the NordSOUND collaboration (Nordic Studies on Occupational and Traffic Noise in Relation to Disease), https://www.cancer.dk/nordsound. The recruitment characteristics for each of the cohorts are described in detail in Table S1; http://links.lww.com/EE/A291. One cohort was from Finland: The FINRISK cohort, which included 8320 participants from the Helsinki/Vantaa and Turku regions.^13^ Five cohorts were from the Stockholm and Uppsala regions in Sweden: The Swedish National Study of Aging and Care in Kungsholmen (SNAC-K), including 3363 participants,^14^ the Screening Across the Lifespan Twin (SALT) study, with 7043 participants,^15^ the Stockholm cohort of 60-year-olds (SIXTY), consisting of 4232 participants,^16^ the Stockholm Diabetes Prevention Programme (SDPP), including 7949 participants,^17^ and the Swedish Mammography Cohort (SMC), consisting of 20,407 women.^18^ Two cohorts were from the Gothenburg region in Sweden: The Swedish Primary Prevention cohort (PPS), consisting of 7495 participants,^19^ and the Gothenburg part of the Multinational Monitoring of Trends and Determinants in Cardiovascular Diseases cohort (GOT-MONICA), with 4875 participants.^20^ One cohort was included from the Malmö area in southern Sweden: The Malmö Diet and Cancer Study (MDC), including 28,098 participants.^21^ Two cohorts were from Denmark: The Diet, Cancer and Health cohort (DCH) recruited in the Copenhagen and Aarhus areas, consisting of 57,053 participants,^22^ and the nationwide Danish Nurses Cohort (DNC), consisting of 28,731 female nurses.^23^ In total, the pooled sample included 177,566 participants with recruitment between 1970 and 2012.

The work in all cohorts was conducted in accordance with national ethical requirements and followed the Helsinki Declaration. Informed consent has been obtained from all cohort participants.

### Outcome assessment

A detailed description of how the outcome data were obtained in the participating cohorts can be found in Table S1; http://links.lww.com/EE/A291. In brief, in eight of the cohorts (DCH, GOT-Monica, PPS, MDC, SDPP, SNAC-K, SIXTY, and FINRISK), height and weight measurements, which were used to calculate BMI, as well as waist circumference measurements were performed by trained personnel at health examinations, while in three cohorts (DNC, SALT, and SMC) data were self-reported. Data on WC were not available in the PPS and SALT cohorts. As outcome variables, we used continuous measures of BMI (calculated from weight [kg] divided by squared height [m^2^]) and WC, as well as categorical, binary measures of overweight (BMI ≥25 kg/m^2^), obesity (BMI ≥30 kg/m^2^),^24^ and central obesity (WC ≥88 cm for women and ≥102 cm for men).^25^

### Exposure assessment

Table S2; http://links.lww.com/EE/A291 describes in detail how exposure to road traffic, railway, and aircraft noise was assessed. For all noise sources, we expressed noise as L_den_, based on the equivalent continuous A-weighted sound pressure level (L_Aeq_) at the most exposed façade, including a penalty of 5 dB for the evening and 10 dB for the night periods. To assess individual exposure, we used information on the participants’ residential history to calculate the time-weighted average noise level 5 years before the baseline of each study, respectively. In the PPS cohort, historic exposure data were lacking before recruitment; therefore, we assumed that the baseline exposure was representative 5 years before baseline (n = 5,146).

All cohorts modeled road traffic noise using the Nordic Prediction Method for road traffic noise.^26^ The model considered address geocodes, screening by buildings and terrain, ground absorption, annual average daily traffic (day/evening/night), distribution of light and heavy vehicles, traffic speed, and road type for all major roads. Traffic information for smaller roads (i.e., roads with <1000 vehicles/day) was available for all cohorts, except the Stockholm cohorts, and information regarding physical noise barriers was available for the Danish, Finnish, and Gothenburg cohorts.

Railway noise was estimated either with the Nordic Prediction Method^26^ or its revised version, Nord2000,^27^ for all cohorts except the SMC, for which railway noise was not modeled. We assessed railway noise at all addresses within a 1,000 m radius around a railway track, metro (Copenhagen and Stockholm), or tram line (Gothenburg and Stockholm). Input variables included geocodes, screening by terrain, barriers, and buildings, average number of trains per period (day/evening/night), train type and speed, and ground absorption. Residences situated more than 1,000 m from a rail, metro, or tramline were considered unexposed to railway noise.

For two of the cohorts (PPS and GOT-MONICA), annual estimates of road traffic and railway noise were generated. For the Danish (DCH and DNC), Uppsala (SMC), and Stockholm cohorts (SDPP, SIXTY, SNAC-K, and SALT), estimates for every 5 years were generated, and for the Malmö cohort (MDC) every 10 years. For FINRISK, noise levels based on the year 2011 were used. For cohorts lacking annual data, we used linear interpolation to estimate noise levels for the intermediate years.

Aircraft noise was estimated in 1 dB categories in the Stockholm cohorts, based on noise maps generated by Swedavia using the Integrated Noise Model.^28^ For FINRISK, aircraft noise for 2011 was modeled in 5 dB categories >50 dB, according to the European Civil Aviation Conference report.^29^ For the Danish cohorts, aircraft noise was estimated in 5 dB categories based on noise maps created by local authorities for separate airports and airfields using the Danish Airport Noise Simulation Model^30^ and the Integrated Noise Model.^28^ Only a limited number of residents were exposed to aircraft noise in the MDC, PPS, GOT-MONICA, and SMC cohorts, and therefore aircraft noise was not estimated. To accommodate differences between cohorts, aircraft noise was categorized.

### Covariates

The selection of covariates was done a priori, based on existing literature and the availability of harmonizable variables across cohorts. Lifestyle variables were obtained from questionnaires filled in by the participants at recruitment, encompassing smoking status (never, former, or current), smoking intensity (among current smokers, grams per day; not available for the PPS cohort), and leisure-time physical activity (“low” as once a month or <1 hour per week, “medium” as about once a week or approximately 1 hour per week, “high” as 3 times a week or more, or >2 hour per week). In addition, information on educational level (“low” as primary school or less, “medium” as up to secondary school or equivalent, or “high” as university degree and more) and marital status (“single” as widowed or never married, or “married,” which also included those living with partner) was obtained from national registers or questionnaires. Area-level mean income, based on small socioeconomically homogeneous areas with ~1,000–2,000 inhabitants, was obtained from registries and categorized in country-specific quartiles.

Long-term air pollution levels were estimated at the residential addresses during the study period using high-resolution dispersion models. Detailed descriptions on the estimation of air pollution for the participating cohorts are presented in Table S3; http://links.lww.com/EE/A291. Air pollution exposure was represented by particulate matter (PM) with an aerodynamic diameter <2.5 μm (PM_2.5_) (available at baseline for all cohorts but PPS) which is predominantly influenced by long-range transport but also by local emissions, and by nitrogen dioxide (NO_2_), primarily reflecting local emissions, such as from road traffic.

### Statistical analyses

Associations between noise exposure and markers of obesity were investigated using linear regression for the continuous outcomes (BMI and WC), estimating beta coefficients (*β*) and 95% confidence intervals (95% CI). Logistic regression was used for the binary outcomes (overweight, obesity, and central obesity), estimating odds ratios (OR) and 95% CI in relation to normal weight participants (BMI<25 or WC below 88 cm and 102 cm for women and men, respectively). Road traffic and railway noise were entered in models as continuous variables, and risk was assessed per 10 dB L_den_. For aircraft noise, the categories ≤40, 40.1–50 dB, and >50 dB L_den_ were used. Road and railway noise values <40 dB were set to 40 dB because of imprecision in low-level noise estimates.

We used two models with increasing levels of adjustment to analyze the associations between transportation noise and the outcomes. Model 1 was adjusted for age, sex, recruitment year, and cohort. The main model, model 2, was additionally adjusted for educational level, marital status, smoking status, physical activity, and area-level income. A complete case analysis approach was applied, thus models 1 and 2 were based on the same analytical sample in all 11 cohorts.

Exposure-response associations were investigated for the binary outcomes, based on clinically relevant cutoffs, using natural splines with 3 degrees of freedom (4 knots placed at p5, p35, p65, and p95 percentiles). For railway noise, because of a skewed distribution, splines were fitted only among participants exposed to noise levels >40 dB L_den_.

Effect modification was investigated by introducing interaction terms between the variable of interest and the noise exposure to model 2 for: sex (women/men), age (<45/45–50/50–55/55–65/>65 years), educational level (low/medium/high), physical activity (low/medium/high), smoking status (never/former/current), PM_2.5_ (quartiles), NO_2_ (quartiles), railway (<54 dB or ≥54 dB), and aircraft (<45 dB or ≥45 dB) noise in the analysis focusing on road traffic noise, and road traffic (<53 dB or ≥53 dB) and aircraft (<45 dB or ≥45 dB) noise in the analysis focusing on railway noise. These cutoffs for noise were set at the health-based guideline values of the WHO.^3^ Furthermore, we also investigated cohort-specific associations by introducing interaction terms between the cohort variable and the exposure variables. The statistical significance of the interaction term was assessed by a Wald test.

In sensitivity analyses, we investigated the effect of further adjustment for smoking intensity, PM_2.5_, NO_2_, and other noise sources (road traffic, railway, and aircraft noise mutually adjusted). We also investigated whether the associations differed depending on the outcome assessment method by stratification on cohorts using measured (DCH, GOT-MONICA, PPS, MDC, SDPP, SNAC-K, SIXTY, and FINRISK) and self-reported obesity (DNC, SALT, and SMC), respectively. Moreover, we investigated the effect of sequential exclusion of the three largest cohorts (DCH, MDC, and DNC) as well as the shape of the exposure-response function after restricting the sample to cohorts using measured outcomes and exclusion of the largest cohort (DCH).

The analyses were performed using Stata 14.1^31^ and R (version 4.1.0).^32^

## Results

All analyses were based on individuals with complete information on transportation noise exposure, outcomes, and covariates in the main model. From the initial sample of 177,566 individuals, this left 162,639 individuals for analysis of road traffic noise and BMI, and 145,281 individuals for railway noise and BMI. Similarly, for the analysis of WC, the analytical sample consisted of 127,040 individuals for road traffic noise and 112,103 individuals for railway noise. Since two of the cohorts included women only (DNC and SMC), the total sample consisted of almost two-thirds of females (65.6%). The median age at recruitment in the sample was 55.0 years, with a range between 46.0 years (GOT-MONICA) and 72.0 years (SNAC-K). The distribution of some other risk factors differed between the cohorts, for instance, educational level, smoking status, physical activity, and area-based income (Table 1).

**Table 1.** Administrative and baseline characteristics of 11 Nordic cohorts^a^

		DCH	DNC	MDC	PPS	GOT- MONICA	SDPP	SIXTY	SNAC-K	SALT	SMC	FINRISK	Total
Recruitment year		1993–1997	1993,1999	1991–1996	1970–1974	1990,1995	1992–1998	1997–1999	2001–2004	1998–2002	1997	1997–2012	1970–2012
Study size		57,053	28,731	28,098	7,495	4,875	7,949	4,232	3,363	7,043	20,407	8,320	177,566
Analytical sample^b^	BMI	54,702	26,327	27,824	5,146	2,565	7,575	3,942	2,559	6,363	17,350	8,320	162,639
	WC	54,696	7,270	27,811	—	2,562	7,565	3,897	—	—	14,930	8,309	127,040
Age (years)	Median	56	50.5	57.8	51.6	46	48	60	72	56	60	51	55
	(5th, 95th percentile)	(50.0, 64.0)	(44.9, 70.9)	(47.1, 71.4)	(47.6, 54.7)	(27.0, 63.0)	(38.0, 54.0)	(60.0, 60.0)	(60.0, 90.0)	(44.0, 79.0)	(50.0, 79.0)	(27.0, 71.0)	(44.9, 71.9)
Sex (%)	Women	52.5	100	60.7	0	52.1	60.7	52.1	61.5	54.8	100	53	65.6
	Men	47.5	0	39.3	100	47.9	39.3	47.9	38.5	45.2	0	47	34.4
Educational level (%)^c^	Low	30.2	0	68	68.2	20.1	31.5	39.6	23.6	26.9	37.9	24.8	33.4
	Medium	46.4	100	17.7	20.5	50.2	38.6	32.3	39.7	36.5	34.1	53.8	47.3
	High	23.4	0	14.3	11.3	29.7	29.9	28	36.8	36.6	28	21.4	19.3
Marital status (%)	Single/ widowers/ never married	23.2	29.6	34.8	14.2	31.5	16.5	25.8	51.6	32.3	23.9	32.4	27.2
	Married/ cohabitants	76.8	70.4	65.2	85.8	68.5	83.5	74.2	48.4	67.7	76.1	67.6	72.8
Smoking status (%)	Never	35.5	34.1	37.9	26.7	48.4	37.2	40.1	44.8	43.5	53.6	44.1	38.6
	Former	28.2	30.7	33.8	33.4	23.3	36.4	38.8	40.1	36.2	23.6	29	30.3
	Current	36.3	35.2	28.3	39.9	28.3	26.4	21.1	15.2	20.3	22.7	26.9	31
Smoking intensity (g nicotine/day)	Median among smokers	15.1	14.4	14	—f	15	15	13	10	10	10	11.2	14.4
Physical activity (%)^d^	Low	51.7	7	51.4	25.3	17.6	65.8	69.2	74.7	54.8	20.5	17.5	39.5
	Medium	19.5	66.4	20.6	59.3	62.5	26.5	23.3	18.5	36.2	57.7	32	35
	High	28.8	26.5	28	15.4	19.9	7.7	7.5	6.7	9	21.8	50.5	25.5
Area-level income (%)^e^	Quartile 1	33	32.9	23.4	25.9	22.3	3.5	4.5	3.1	0.7	25.2	19.8	25.9
	Quartile 2	22.5	26.6	21.1	22.4	15.9	5.6	8.7	0	10.5	22.6	25.9	21.1
	Quartile 3	16.9	24.8	26.7	24.6	21.8	21	24.2	0.2	18.5	27.1	26.5	21.9
	Quartile 4	27.5	15.7	28.8	27.1	40	69.8	62.6	96.7	64	25.2	27.9	31.1

a DCH, DNC, MDC, PPS, GOT-MONICA, SDPP, SIXTY, SNAC-K, SALT, SMC, FINRISK.

b Analytical samples based on the main model, Model 2 (nonmissingness in cohort, sex, age, calendar year, educational level, marital status, smoking status, physical activity, and area income); SMC is not included in the railway analysis due to the absence of exposure data.

c Low (primary school or less), medium (up to secondary school or equivalent), high (university degree and more).

d Low (once a month or less, <1 hour/week), medium (about once a week, ~1 hour/week), high (3× a week or more, >2 hours/week).

e Mean household income in the area where the participant lived at baseline, in quartiles for each country.

f —, no data.

BMI indicates body mass index; WC indicates waist circumference;DCH, Diet, Cancer and Health cohort; DNC, Danish Nurses Cohort; FINRISK, the National FINRISK study; GOT-MONICA, Multinational Monitoring of Trends and Determinants in Cardiovascular Disease cohort (Gothenburg); MDC, Malmö Diet and Cancer study; PPS, Primary Prevention Study cohort; SALT, Stockholm Screening Across the Lifespan Twin study; SDPP, Stockholm Diabetes Prevention Program; SIXTY the Stockholm Cohort of 60-year-olds; SMC, The Swedish Mammography Cohort; SNAC-K, Swedish National Study of Aging and Care in Kungsholmen.

The median 5-year time-weighted average road traffic noise level before baseline was 55.0 dB L_den_, with cohort-specific medians ranging from 44.4 dB in the SDPP cohort to 62.3 dB in the SNAC-K cohort (Table 2). Pooled and cohort-specific distributions of road traffic noise exposure, displayed in Figures S1 and S2; http://links.lww.com/EE/A291, revealed that some cohorts contribute only marginally to the exposure above 60 dB L_den_, including a majority of the Stockholm cohorts. Approximately a quarter (25.4%) of participants were exposed to railway noise above 40 dB L_den_ with a median of 50.3 dB L_den_ within the 10 cohorts with available data (Table 2 and Figure S3; http://links.lww.com/EE/A291). Exposure to aircraft noise was of relevance in seven of the 11 cohorts, but only few individuals (7% of total sample) were exposed to >40 dB L_den_ (Table 2 and Figure S4; http://links.lww.com/EE/A291).

**Table 2.** 5-year time-weighted average exposure to transportation noise and air pollution in 11 Nordic cohorts^a^

Exposure		DCH	DNC	MDC	PPS	GOT-MONICA	SDPP	SIXTY	SNAC-K	SALT	SMC	FINRISK	Total
Road traffic noise, continuous (dB L_den_)	Median	56.7	53.5	54.6	57.5	55.7	44.4	50.7	62.3	51.7	55.7	54.1	55.0
	(5th, 95th percentile)	(45.0, 69.3)	(40.2, 66.9)	(42.4, 67.6)	(45.4, 72.3)	(40.0, 69.7)	(40.0, 58.2)	(40.0, 66.6)	(51.4, 71.8)	(40.0, 67.0)	(40.3, 66.5)	(42.0, 68.1)	(40.1, 68.1)
Railway noise, categorical (%)	Exposed >40 dB	25.9	18.9	28.1	15.2	19.2	14.4	31.8	52.0	34.3	—b	34.2	25.4
Railway noise, continuous (dB L_den_)	Median	52.3	53.1	46.8	44.8	44.6	50.5	48.9	49.0	49.2	—	47.6	50.3
	(5th, 95th percentile)	(42.2, 66.6)	(42.0, 66.0)	(40.6, 67.7)	(40.4, 58.8)	(40.4, 57.6)	(42.2, 67.3)	(40.7, 63.7)	(41.7, 59.3)	(40.7, 64.1)	—	(40.7, 63.7)	(41.1, 65.8)
Aircraft noise, categorical (%)	<40 dB	98.6	98.8	—	—	—	74.7	83.5	15.8	83.3	—	95.6	93.3
	40–49 dB	0.7	0.4	—	—	—	9.6	13.5	65.0	13.6	—	0.6	3.9
	50–54 dB	0.04	0.5	—	—	—	10.3	2.3	18.4	2.8	—	2.4	1.8
	≥55 dB	0.7	0.4	—	—	—	45.4	0.7	0.9	0.4	—	1.4	1.0
PM_2.5_, continuous (µg/m^3^)^c^	Median	19.3	21.0	11.1	—	10.0	7.6	8.1	8.3	7.7	14.0	7.4	15.8
	(5th, 95th percentile)	(18.6, 24.0)	(15.7, 26.4)^e^	(9.7, 12.5)	—	(8.1, 12.0)	(6.7, 8.4)	(6.9, 9.6)	(7.7, 10.3)	(6.5, 9.3)	(12.8, 15.2)	(6.8, 9.3)	(7.2, 24.0)
NO_2_, continuous (µg/m^3^)^d^	Median	27.4	10.9	24.4	30.8	27.4	8.6	12.9	20.4	13.1	6.5	15.4	21.7
	(5th, 95th percentile)	(19.5, 46.6)	(5.6, 28.2)^f^	(13.8, 35.2)	(23.1, 43.4)^g^	(18.5, 42.0)	(4.9, 13.9)	(5.3, 26.1)	(15.1, 32.3)	(5.5, 25.4)	(3.8, 17.4)	(10.9, 25.1)	(5.2, 38.9)

a DCH, DNC, MDC, PPS, GOT-MONICA, SDPP, SIXTY, SNAC-K, SALT, SMC, FINRISK.

b —, no data.

c PM_2.5_, particulate matter with an aerodynamic diameter of ≤2.5 µm (fine particulate matter).

d NO_2_, nitrogen dioxide.

e 2.5% missing.

f 9.5% missing.

g 2.4% missing.

DCH indicates Diet, Cancer and Health cohort; DNC, Danish Nurses Cohort; FINRISK, the National FINRISK study; GOT-MONICA, Multinational Monitoring of Trends and Determinants in Cardiovascular Disease cohort (Gothenburg); MDC, Malmö Diet and Cancer study; PPS, Primary Prevention Study cohort; SALT, Stockholm Screening Across the Lifespan Twin study; SDPP, Stockholm Diabetes Prevention Program; SIXTY the Stockholm Cohort of 60-year-olds; SMC, The Swedish Mammography Cohort; SNAC-K, Swedish National Study of Aging and Care in Kungsholmen.

The median exposure to PM_2.5_ among the 10 cohorts with available data was 15.8 µg/m^3^ and showed a tendency of a downward south-to-north gradient (Table 2, Figure S5; http://links.lww.com/EE/A291), with generally higher exposure levels in the Danish cohorts and lower in the Finnish and Stockholm-based cohorts (Figure S6; http://links.lww.com/EE/A291). One exception, however, is the SMC, which had a relatively high median PM_2.5_ exposure (14.0 µg/m^3^) for its (northern) location. The median NO_2_ was 21.7 µg/m^3^ (Table 2 and Figure S5; http://links.lww.com/EE/A291) and tended to be higher in urban cohorts compared with the more rural (Figure S7; http://links.lww.com/EE/A291).

The Spearman rank correlation coefficients between road traffic noise, railway noise, PM_2.5_, and NO_2_ were very weak to moderate. The highest correlation was observed for road traffic noise and NO_2_ (R_s_ = 0.56) (Table S4; http://links.lww.com/EE/A291), with cohort-specific correlations ranging from 0.39 in the SMC and 0.72 in the SIXTY cohorts.

The median BMI within the study population was 24.9 kg/m^2^, varying from 23.1 in the DNC to 26.2 in the SIXTY cohort (Table 3). In total, 48.8% of the study participants were classified as overweight or obese (BMI ≥25 kg/m^2^). Correspondingly, the proportion of individuals classified as obese (BMI ≥30 kg/m^2^) was 12% in the total sample and ranged from 5.6% in the DNC to 19.0% in the SIXTY cohort. Among individuals of the nine cohorts with available data on WC, the median WC was 80.0 cm in women and 94.0 cm in men. Central obesity, defined as ≥88 cm in women and ≥102 cm in men, was identified in 24.9% of these participants, ranging from 17.8% (MDC) to 37.6% (SALT) in the different cohorts.

**Table 3.** Measures of overweight and obesity at baseline in 11 Nordic cohorts^a^

Outcome		DCH	DNC	MDC	PPS	GOT- MONICA	SDPP	SIXTY	SNAC-K	SALT	SMC	FINRISK	Total
BMI (kg/m^2^), continuous	N	54,702	26,327	27,824	5,146	2,565	7,541	3,942	2,559	6,363	17,350	8,320	162,639
	Median	25.5	23.1	25.3	25.1	24.5	25.1	26.2	25.4	24.2	24.3	25.8	24.9
	(5th, 95th percentile)	(20.4, 33.4)	(19.2, 30.2)	(20.2, 33.0)	(20.7, 30.5)	(19.7, 32.3)	(20.4, 33.3)	(21.0, 34.3)	(19.9, 32.8)	(19.6, 30.6)	(19.7, 32.0)	(20.2, 35.0)	(19.9, 32.7)
BMI (kg/m^2^), categorical (%)	<24.9	43.8	71.5	46.7	48.3	55.9	48.2	36.4	45.5	60.2	57.4	42.1	51.2
	25–29.9	41.6	22.9	39.8	45.2	34.3	39	44.6	41.3	33.3	32.5	38.9	36.8
	≥30	14.6	5.6	13.5	6.5	9.8	12.8	19	13.2	6.5	10.1	18.9	12
WC (cm), continuous	Women												
	N	28,697	7,270	16,873	—b	1,333	4,594	2,026	—	—	14,930	4,375	80,098
	median	80	80	76	—	76	79	85	—	—	82	81.5	80
	(5th, 95th percentile)	(67.0, 103.0)	(68.0, 101.0)	(64.0, 98.0)	—	(65.0, 98.0)	(67.0, 100.0)	(70.0, 109.0)	—	—	(69.0, 102.0)	(67.0, 108.5)	(66.0, 102.0)
	Men												
	N	25,999	—	10,938	—	1,229	2,971	1,871	—	—	—	3,934	46,942
	median	95	—	93.0	—	91	92	97	—	—	—	95	94
	(5th, 95th percentile)	(81.0, 114.0)	—	(79.0, 111.0)	—	(77.0, 107.0)	(80.0, 108.0)	(82.0, 116.0)	—	—	—	(78.5, 116.5)	(80.0, 113.0)
Central obesity, binary^c^	N (%)	14,407 (26.3)	1,798 (24.7)^d^	4,944 (17.8)	—	391 (15.3)	1,381 (18.2)	1,470 (37.6)	—	—	4,644 (31.1)^d^	2,573 (31.0)	31,608 (24.9)

Based on analytical samples of N = 162,673 for BMI-based outcomes and N = 127,088 for WC and central obesity.

a DCH, DNC, MDC, PPS, GOT-MONICA, SDPP, SIXTY, SNAC-K, SALT, SMC, FINRISK.

b —, no data.

c Women: ≥88 cm, men: ≥102 cm.

d Including women only.

BMI indicates body mass index; WC, waist circumference; DCH indicates Diet, Cancer and Health cohort; DNC, Danish Nurses Cohort; FINRISK, the National FINRISK study; GOT-MONICA, Multinational Monitoring of Trends and Determinants in Cardiovascular Disease cohort (Gothenburg); MDC, Malmö Diet and Cancer study; PPS, Primary Prevention Study cohort; SALT, Stockholm Screening Across the Lifespan Twin study; SDPP, Stockholm Diabetes Prevention Program; SIXTY the Stockholm Cohort of 60-year-olds; SMC, The Swedish Mammography Cohort; SNAC-K, Swedish National Study of Aging and Care in Kungsholmen.

Table 4 shows associations of road traffic and railway noise, respectively, with markers of obesity in relation to the time-weighted average exposure 5 years before baseline. Based on the main model (Model 2), we found associations between road traffic as well as railway noise exposure and all outcomes. For road traffic, the OR for obesity was 1.06 (95% CI = 1.03, 1.08) per 10 dB L_den_, and for central obesity 1.03 (95% CI = 1.01, 1.05). Corresponding ORs for railway noise were 1.06 (95% CI = 1.03, 1.09) and 1.06 (95% CI = 1.04, 1.08), respectively. We did not find aircraft noise associated with higher *β* coefficients or ORs of any of the obesity markers (Table S5; http://links.lww.com/EE/A291).

**Table 4.** Beta coefficients (b) and odds ratios (OR) in relation to the 5-year time-weighted average road traffic and railway noise exposure, respectively, and markers of obesity in pooled analyses of 11 Nordic cohorts^a^

	N/sample	n/cases	Model 1^b^	Model 2 (main model)^c^
Road traffic noise				
Linear regression, *β* (95% CI) per 10 dB L_den_				
BMI (kg/m^2^)	162,639	—	0.04 (0.01, 0.06)	0.05 (0.03, 0.08)
WC (cm)	127,040	—	0.09 (0.02, 0.17)	0.08 (0.00, 0.16)
Logistic regression, OR (95% CI) per 10 dB L_den_				
Overweight (BMI ≥25 vs BMI<25)	162,639	79,367	1.01 (1.00, 1.02)	1.03 (1.01, 1.04)
Obesity (BMI≥30 vs BMI<25)	102,823	19,584	1.06 (1.03, 1.08)	1.06 (1.03, 1.08)
Central obesity^d^	127,040	31,608	1.04 (1.02, 1.06)	1.03 (1.01, 1.05)
Railway noise^e^				
Linear regression, *β* (95% CI) per 10 dB L_den_				
BMI (kg/m^2^)	145,281	—	0.09 (0.06, 0.12)	0.08 (0.04, 0.11)
WC (cm)	112,103	—	0.31 (0.21, 0.41)	0.23 (0.14, 0.33)
Logistic regression, OR (95% CI) per 10 dB L_den_				
Overweight (BMI ≥25 vs BMI<25)	145,281	70,897	1.04 (1.02, 1.05)	1.03 (1.02, 1.05)
Obesity (BMI≥30 vs BMI<25)	91,103	17,822	1.07 (1.04, 1.10)	1.06 (1.03, 1.09)
Central obesity^d^	112,103	26,963	1.08 (1.05, 1.10)	1.06 (1.04, 1.08)

a DCH, DNC, MDC, PPS, GOT-MONICA, SDPP, SIXTY, SNAC-K, SALT, SMC, FINRISK.

b Model 1: adjusted for age, sex, recruitment year, and cohort.

c Model 2 (main model):additionally adjusted for educational level, marital status, smoking status, physical activity, and area-level income.

d Central obesity: momen: WC ≥88 cm, men: WC ≥102 cm.

e SMC cohort is not included in the analysis due to absence of exposure data.

BMI indicates body mass index; WC, waist circumference; DCH, Diet, Cancer and Health cohort; DNC, Danish Nurses Cohort; FINRISK, the National FINRISK study; GOT-MONICA, Multinational Monitoring of Trends and Determinants in Cardiovascular Disease cohort (Gothenburg); MDC, Malmö Diet and Cancer study; PPS, Primary Prevention Study cohort; SALT, Stockholm Screening Across the Lifespan Twin study; SDPP, Stockholm Diabetes Prevention Program; SIXTY the Stockholm Cohort of 60-year-olds; SMC, The Swedish Mammography Cohort; SNAC-K, Swedish National Study of Aging and Care in Kungsholmen.

**Figure 1.**
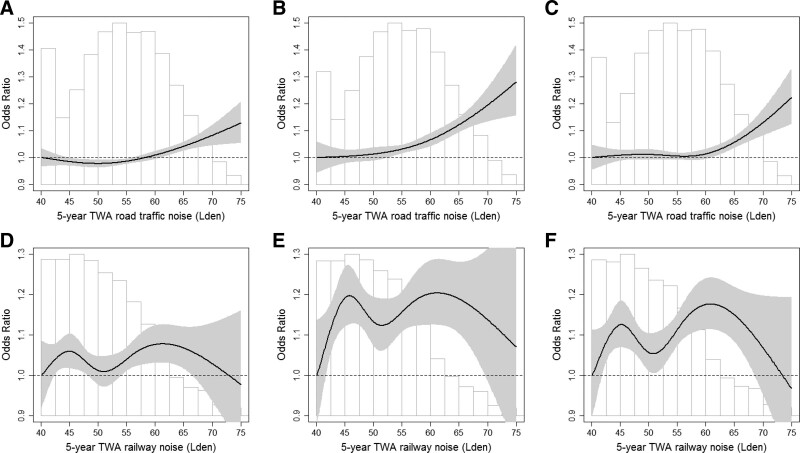
Exposure-response associations (OR per 10 dB increment) between road traffic and railway noise, respectively, and overweight, obesity, and central obesity in pooled analyses of 11 Nordic cohorts. Road traffic noise (A–C): natural splines with knots at the 20th, 40th, 60th, and 80th percentile. A, Overweight: N = 162,639; n = 79,367 cases of overweight. Likelihood ratio test of linear vs spline: *P* value = 0.0387. In a separate analysis OR over 53 dB as an inflection point is OR 1.064 (95% CI = 1.031, 1.084) per 10 dB increase. B, Obesity: N = 102,823; n = 19,584 cases of obesity. Likelihood ratio test of linear vs spline: *P* value = 0.0188. In a separate analysis OR over 53 dB as an inflection point is 1.102 (95% CI = 1.055, 1.151) per 10 dB increase. C, Central obesity: N = 127,040; n = 31,608 cases of central obesity. Likelihood ratio test of linear vs spline: *P* value = 0. 0052. In a separate analysis OR over 58 dB as an inflection point is 1.086 (95% CI = 1.011, 1.166) per 10 dB increase. Railway noise (D–F): D, Overweight: natural splines with the forced placement of knots at 44.1, 48.1, 52.4, and 57.6 dB L_den_ (the 20th, 40th, 60th, and 80th percentile among those exposed to railway noise >40 dB). N = 36,747, n = 18,989 cases. E, Obesity: natural splines with the forced placement of knots at 44.2, 48.2, 52.5, 57.6 dB L_den_ (the 20th, 40th, 60th, and 80th percentile among exposed to railway noise >40 dB). N = 22,770, n = 5,012 cases. F, Central obesity: natural splines with the forced placement of knots at 44.1, 48.0, 52.4, 57.7 dB L_den_ (the 20th, 40th, 60th, and 80th percentile among exposed to railway noise >40 dB). N = 28,944, n = 7,475 cases. Adjusted for cohort, sex, age, recruitment year, educational level, marital status, area income, smoking status, and physical activity. Overweight: body mass index (BMI) ≥25 kg/m² compared with BMI <25 kg/m². Obesity: BMI ≥30 kg/m² compared with BMI <25 kg/m². Central obesity: women: ≥88 cm compared with <88 cm; men: ≥102 cm compared with <102 cm. TWA, time-weighted average.

The shape of the exposure-response associations for road and railway noise in relation to obesity and central obesity, modeled using natural splines, is shown in Figure 1. For road traffic noise, we found indications of a threshold around 50–55 dB L_den_ for obesity and around 55–60 dB L_den_ for central obesity, with ORs above the thresholds of 1.10 (95% CI = 1.06, 1.15) and 1.09 (95% CI = 1.01, 1.17) per 10 dB L_den_, respectively. For railway noise, a departure from linearity was indicated for the exposure-response functions, for obesity as well as central obesity, but with no apparent thresholds.

**Figure 2.**
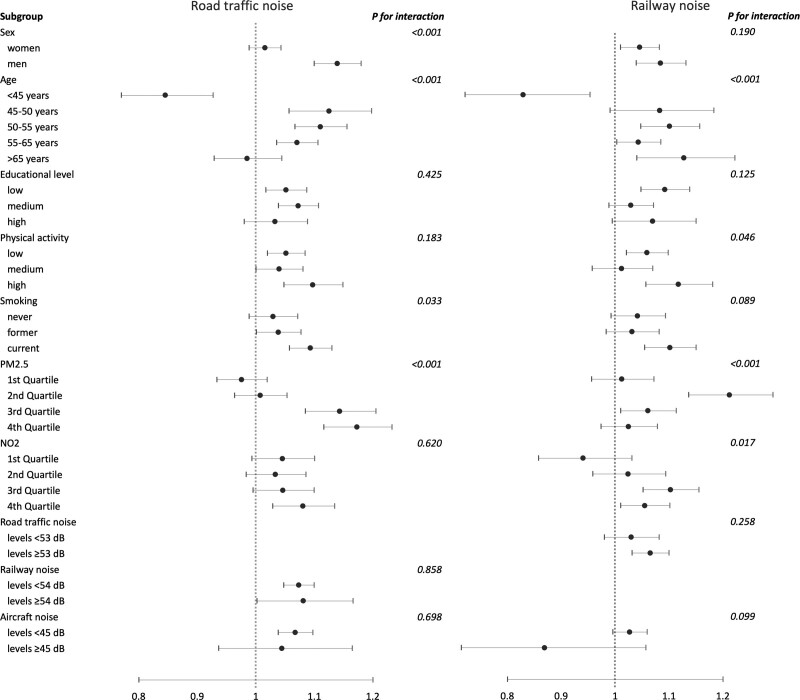
Obesity in relation to road traffic and railway noise exposure 5 years before baseline (OR and 95% CI per 10 dB L_den_) according to different characteristics of the study subjects in pooled analyses of 11 Nordic cohorts. Adjusted for cohort, sex, age, recruitment year (<1995, 1995–2000, >2000), educational level, marital status, area income, smoking status, and physical activity. Analytical samples differ for analysis with different interactors for road traffic and railway noise.

In interaction analyses, we investigated the effect modification of several risk factors regarding the associations between road traffic and railway noise, respectively, and obesity (Figure 2) as well as overweight and central obesity (Figure S8–S9; http://links.lww.com/EE/A291). For road traffic noise and obesity, interactions were indicated for sex, age, smoking, and exposure to PM_2.5_, with stronger associations in men, current smokers, and participants exposed to higher levels of PM_2.5_. For instance, individuals within the highest quartile of PM_2.5_ exposure appeared to have a particularly high risk of obesity in relation to road traffic noise exposure, with an OR of 1.17 (95% CI = 1.12, 1.23) per 10 dB L_den_. Furthermore, individuals younger than 45 years appeared to have a reduced risk of obesity in relation to road traffic noise exposure in comparison to all other age groups. Similar patterns of interactions were seen also for overweight and central obesity (Figure S8–S9; http://links.lww.com/EE/A291), however, with a suggested interaction also for physical activity for overweight and no interaction for smoking but potentially for NO_2_ concerning central obesity. For railway noise, there were indications of interaction with age, physical activity, smoking, and PM_2.5_ exposure (Figure 2 and Figure S8-S9; http://links.lww.com/EE/A291).

Results from the sensitivity analyses, focusing on overweight, obesity, and central obesity, are presented in Tables S6 and S7; http://links.lww.com/EE/A291 for road traffic and railway noise, respectively. For road traffic noise, additional adjustment for smoking intensity, PM_2.5_, and NO_2_ or adjustment of exposure to other noise sources (i.e., railway or aircraft noise) did not change the ORs for any of the outcomes. However, we observed associations only in cohorts using measurements, not in those using self-reported outcome data. For example, the OR for obesity among individuals with self-reported data was 0.98 (95% CI = 0.94, 1.03) per 10 dB L_den_, but 1.09 (95% CI = 1.06, 1.12) in those with measurements. Furthermore, in analyses sequentially leaving out one of the three largest cohorts (DCH, MDC, and DNC), the associations for all outcomes were reduced to unity when excluding DCH for road traffic noise (Table S6; http://links.lww.com/EE/A291). Restricting the analyses of exposure-response functions to cohorts with measured outcomes indicated associations for overweight, obesity, and central obesity, although with no clear thresholds, but following additional exclusion of DCH only the association with central obesity remained (Figure S10; http://links.lww.com/EE/A291).

For railway noise, the additional adjustments for smoking intensity, NO_2_, PM_2.5,_ and other noise sources (i.e., road traffic and aircraft noise) did not change the risk estimates (Table S7; http://links.lww.com/EE/A291). However, we observed the same pattern as for road traffic noise regarding measured versus self-reported assessment of BMI and WC, suggesting associations only in individuals with measured outcomes. Following the sequential exclusion of the three largest cohorts, the observed associations for railway noise remained also after the exclusion of the DCH.

Cohort-specific results for overweight, obesity, and central obesity are presented in Figures S11 and S12; http://links.lww.com/EE/A291 for road traffic and railway noise, respectively. The results for road traffic noise indicate heterogeneity between the cohorts with generally more pronounced positive associations in the Danish cohorts and the MDC, the most southern cohorts, and to some extent also the SDPP and the PPS cohorts. On the other hand, we also observed inverse associations, for example, for the SMC. For railway noise, there was also some heterogeneity between the studies with two cohorts indicating consistent associations (DCH and MDC), but less clear associations in the remaining cohorts.

## Discussion

The results of this large cross-sectional study, pooling data from 11 Nordic cohorts including more than 160,000 individuals, indicate associations between long-term exposure to both road traffic and railway noise, and prevalence of obesity. For road traffic noise, although mainly driven by the largest cohort (DCH), we observed exposure-response associations with indications of thresholds around 50–55 and 55–60 dB L_den_ for obesity and central obesity, respectively. A particularly strong association was found among participants with concurrent exposure to PM_2.5_. Similar associations as for road traffic noise were also detected for railway noise, but with no apparent thresholds. Aircraft noise was not associated with any of the obesity markers; however, the study suffered from a low number of highly exposed, making risk estimates uncertain.

There is growing evidence that transportation noise may affect obesity markers, but most previous studies have focused on road traffic noise, and there are still uncertainties regarding the shape of the associations.^4^ Only a few studies have been conducted with cohorts outside of the present study. One cross-sectional analysis of a Swiss cohort found increases in measured BMI of 0.39 kg/m^2^ (95% CI = 0.18, 0.59) and in measured WC of 0.93 cm (95% CI = 0.37, 1.50) per 10 dB in 5-year mean exposure to road traffic noise.^33^ A corresponding change in the noise levels was also associated with obesity (OR = 1.17, 95% CI = 1.03, 1.33), overweight (OR = 1.20, 95% CI = 1.08, 1.33), and central obesity (OR = 1.16, 95% CI = 1.04, 1.29). Another study including three cohorts from the Netherlands, Norway, and the UK, based on measured BMI and WC, found that in the UK cohort, a 10 dB L_den_ higher annual mean road traffic noise exposure was associated with an increase in BMI of 0.14 kg/m^2^ (95% CI = 0.11, 0.18) and WC of 0.27 cm (95% CI = 0.19, 0.35), while no associations were found in the other two cohorts.^34^ A higher prevalence of obesity (OR = 1.06, 95% CI = 1.04, 1.08) and central obesity (OR = 1.05, 95% CI = 1.04, 1.07 per 10 dB L_den_) was also observed in the UK cohort. We found associations of 0.05 kg/m^2^ (95% CI = 0.03, 0.08) for BMI and of 0.08 cm (95% CI = 0.00, 0.16) for WC as well as ORs for obesity and central obesity of 1.06 (95% CI = 1.03, 1.08) and 1.03 (95% CI = 1.01, 1.05 per 10 dB L_den_), respectively. Our estimates appear substantially lower than in the Swiss and UK cohorts, except for the ORs for obesity and central obesity in the UK cohort. The reasons behind the differences in estimates are unclear but factors of importance may be differences in exposure levels, characteristics of the study populations, differences and accuracy of the noise estimation methods, interacting exposures, and varying building techniques.

We found that road and railway noise was only associated with high odds of obesity markers in the sample of cohorts with objectively measured BMI/WC, whereas pooled estimates based on the three cohorts relying only on self-reported BMI/WC (DNC, SALT, and SMC) were close to unity. Previous research has shown that BMI derived from self-reported height and weight tends to underestimate the prevalence of overweight and obesity.^35^ Thus, the use of self-reported and self-measured anthropometrics in three of the participating cohorts may have led to misclassification of the outcome, potentially affecting the validity of our results by diluting the associations. An alternative explanation could be that two of these three cohorts included only women, for which we observed weaker associations in comparison to men.

None of the two previous studies (described above) comprehensively investigated the shape of the exposure-response functions. However, in both the UK and Norwegian cohorts, the associations appeared strongest in the highest exposure category, that is, above 55 dB L_den_.^34^ In the present study, we observed thresholds of around 50–55 and 55–60 dB L_den_ for road traffic noise in relation to the prevalence of obesity and central obesity, respectively. Above these thresholds, we found evidence of exposure-response associations with estimated risk increases of approximately 10% per 10 dB L_den_ increment. However, restricting the sample to cohorts using measured outcomes only, no apparent thresholds were indicated. It should also be noted that after excluding the largest cohort (DCH), the associations for overweight/obesity, but not central obesity, reduced to unity, which points to a considerable influence by this cohort. In largely the same study base as in our study, a threshold at around 55 dB L_den_ was observed in the association between road traffic noise exposure and the risk of ischemic heart disease,^5^ but no threshold was apparent for stroke.^36^ Thresholds in the exposure-response relationships have profound effects for the health impact assessment and should be investigated in future studies on transportation noise exposure and adiposity markers.

Only one previous study, not including the cohorts in our pooled analysis, has investigated exposure to railway or aircraft noise exposure and obesity.^33^ This study did not find clear associations between either of the two noise sources and any of the obesity markers. In contrast, we observed associations between railway noise exposure and all obesity markers, although the findings are more uncertain than for road traffic noise due to a smaller sample. It should be noted that our risk estimates were close to those in the Swiss study. Similar to the Swiss study, we did not see associations between aircraft noise exposure and any of the obesity markers, but only a few participants were exposed to high noise levels. More studies are needed on railway or aircraft noise and obesity markers, which preferably should be conducted in areas close to these noise sources. Generally, both railway and aircraft noise exposure are less common in cohorts based on the general population, making risk estimates uncertain.

Overall, there is limited evidence on the interactions of air pollution and traffic noise regarding disease etiology of overweight and obesity. Previous research on the impact of ambient air pollution on markers of obesity indicates mixed results. A systematic review by An et al^37^ found that 29 (44%) of 66 reported associations, air pollutants such as PM, NO_2_, SO_2_, O_3_, and overall air quality were positively associated with body weight, 29 (44%) showed null findings and 8 (12%) indicated inverse associations.^37^ Proposed mechanisms for the association of air pollution and adiposity include increased oxidative stress, systemic inflammation and adipose tissue inflammation, elevated risk for chronic comorbidity, and insufficient physical activity. In our study, we generally found higher risk estimates among individuals who had both high road traffic noise levels and high PM_2.5_ exposure. This may indicate an interaction; however, it could also be due to the more prevalent concurrent exposure of both noise and air pollution in the Danish cohorts. Few previous studies have investigated the impact of air pollution and traffic noise on obesity markers simultaneously and noise is generally not controlled for in studies of air pollution and obesity. However, a study based on the UK Biobank, found positive associations between air pollution and adiposity with minimal confounding by traffic noise.^38^ Clearly, additional studies are needed to clarify the influence of combined exposure to traffic noise and air pollution on various markers of obesity.

We also observed stronger associations in men than in women, in current smokers as compared with never and former smokers, and among individuals with a high level of physical activity compared with those less active. Furthermore, young individuals (<45 years) had a lower risk of obesity in relation to road traffic noise than older ones. Our results of a particularly strong association in males conflict with findings by Foraster et al^33^ who did not find any difference according to sex. They are also contradicted by results from a newly conducted cross-sectional investigation of perceived traffic noise in the bedroom and self-measured WC and BMI where associations were found solely in women.^39^ A Norwegian study with objectively measured obesity markers also found an association among highly noise-sensitive women only.^40^ There are sex differences in obesity etiology^41^ and although our results regarding women appear to be driven by the SMC, a female cohort using self-reported outcome data, they motivate additional research investigating the sex-specific influence of traffic noise on obesity. While the higher risk estimates among smokers may be explained by mechanisms analogous to those for air pollution, the greater risk in participants with a high physical activity could potentially be explained by higher exposure to noise and air pollution during exercise (if performed outdoors in the vicinity of the residence). The reason for the inverse associations observed in young individuals is unclear but may be due to a general higher prevalence of overweight and obesity in older age, a shorter duration of exposure, residual confounding, or could be a chance finding.

The cohort-specific analyses indicated a relatively large heterogeneity in our data with generally higher risk estimates in the Danish cohorts and in the MDC, where the DCH clearly influenced the results for road traffic noise. One potential explanation could be the higher levels of PM_2.5_ in these cohorts, interacting with road traffic noise. Although pooling of data has obvious advantages, there are also many challenges of combining data from different cohorts, for example, relating to data quality and harmonization, which may influence the interpretation of our results. First, due to differences in the numbers of highly exposed in the study populations, the thresholds in the exposure-response relation could contribute to explaining the heterogeneity in associations between the cohorts included in our study. Second, the use of self-reported obesity measures in three of the cohorts may have underestimated the prevalence of overweight and obesity in these cohorts and led to attenuation of the associations. Third, there were also differences between the cohorts regarding the estimation of traffic noise exposure. Although all cohorts used the same or similar noise calculation methods to assess source-specific exposure to noise, there were differences in the quality of input data, which may be of importance for the validity of the estimations. For instance, the cohorts from Stockholm lacked information on traffic flows on smaller roads (<1000 vehicles/day) which may have led to an underestimation of the exposure for individuals in these cohorts. Also, while most cohorts accounted for screening by buildings, only four accounted for screening by noise barriers. Furthermore, the inclusion of apartment floor, used to assess calculation height, may have led to a higher accuracy of noise estimations in the Danish cohorts, resulting in less attenuation of risk estimates related to exposure misclassification. Fourth, there were also differences in the estimation methods regarding air pollution. While there was a general trend of a downward south-to-north gradient in PM_2.5_ exposure, the estimates for SMC (localized in the Region of Uppsala, Sweden) were higher than anticipated. Presumably, this could be due to methodological differences in the air pollution modeling, for example, assumptions of the proportion of PM_2.5_ in wear particles from studded tires and calculation methods for particle emissions from household wood burning.

The strengths of this study include its size and combination of data from 11 different cohorts based on Nordic populations (Danish, Finnish, and Swedish), which enabled high-precision estimates of the association between traffic noise and markers of obesity. Another strength of this study is the inclusion of several different markers of obesity, measuring both general and central obesity, and the consistency of associations across these. Furthermore, all cohorts used objective methods for assessment of noise exposure, considering the individuals’ residential history 5 years before baseline. Based on these data, we were able to assess exposure-response functions for both road traffic and railway noise. Using questionnaire and register data, we were also able to control for several important lifestyle and socioeconomic variables and to investigate their interactive effects with noise exposure. However, there were few participants highly exposed to aircraft noise within the participating cohorts, which led to a low exposure contrast and uncertain estimates of association with health outcomes. Another limitation is the lack of longitudinal information regarding the markers of obesity, which limits the interpretation of causal associations because of the unclear time sequence between exposure and health outcomes. Finally, due to the limitations of data harmonization, residual confounding cannot be excluded.

In conclusion, this study indicates associations between long-term exposure to both road traffic and railway noise, and prevalence of obesity as well as central obesity. For road traffic noise, we observed thresholds at around 50–55 dB and 55–60 dB L_den_ for obesity and central obesity, respectively, with an approximate 10% risk increase per 10 dB L_den_ thereafter in the full sample. However, sensitivity analyses indicated associations only in cohorts using measured outcomes, with no apparent thresholds, and a strong influence by the largest cohort. Overall, our findings point to a potential pathway between transportation noise and cardiometabolic disease.

## Acknowledgments

*The authors wish to express their gratitude to all individuals participating within the cohorts, without which this study would not have been feasible. Further, the authors thank the Swedish Twin Registry for providing access to data regarding the SALT cohort.*

## Supplementary Material
