# Supplementary material for: Long-term exposure to transportation noise and obesity: A pooled analysis of eleven Nordic cohorts
Source: Environ Epidemiol. 2024 Jul 8;8(4):e319. doi: 10.1097/EE9.0000000000000319 (PMC11233097; doi:10.1097/EE9.0000000000000319)
Supplement: Supplementary file 1 [file ee9-8-e319-s001.pdf]

Supplementary Material

Long-term exposure to transportation noise and obesity – A pooled analysis of eleven Nordic cohorts

Åsa Persson et al.

Table S1: Detailed information on the participating cohorts and outcome assessment method. 3

Table S2: Detailed information on estimation of road traffic, railway, and aircraft noise for the participating cohorts. 6

Table S3: Detailed information on estimation of air pollution for the participating cohorts. 11

Table S4: Spearman correlation coefficients between road traffic noise, railway noise, $PM_{2.5}$ and NO_2. 13

Table S5: Pooled and cohort-specific results for aircraft noise. 14

Table S6: Sensitivity analyses for road traffic noise in relation to overweight, obesity and central obesity (OR and 95% CI per 10 dB L_{den} increase). 15

Table S7: Sensitivity analyses for railway noise in relation to overweight, obesity and central obesity (OR and 95% CI per 10 dB L_{den} increase). 16

Figure S1: Histograms of road traffic, railway, and aircraft noise exposure for the pooled cohort (displaying levels of ≥40 dB L_{den} only). 17

Figure S2: Cohort-specific histograms of exposure to road traffic noise (displaying levels of ≥40 dB L_{den} only). 18

Figure S3: Cohort-specific histograms of exposure to railway noise (displaying levels of ≥40 dB L_{den} only). 19

Figure S4: Cohort-specific histograms of exposure to aircraft noise (displaying levels of ≥40 dB L_{den} only). Data only available in 5 dB for DCH, DNC and FINRISK. 20

Figure S5: Histograms of air pollution exposure ($PM_{2.5}$, NO_2) for the pooled sample. 21

Figure S6: Cohort-specific histograms of exposure to $PM_{2.5}$. 22

Figure S7: Cohort-specific histograms of exposure to NO_2. 23

Figure S8: Overweight in relation to road traffic and railway noise exposure 5 years prior to baseline (OR and 95% CI per 10 dB L_{den}) according to different characteristics of the study subjects. 24

Figure S9: Central obesity in relation to road traffic and railway noise exposure 5 years prior to baseline (OR and 95% CI per 10 dB L_{den}) according to different characteristics of the study subjects. 26

Figure S10: Exposure-response associations between road traffic noise and overweight, obesity and central obesity, respectively, based on cohorts with measured outcomes only and additionally excluding the DCH in the lower panels. 28

Figure S11: Cohort-specific results performed through interaction analyses for overweight, obesity and central obesity in relation to road traffic noise (OR and 95% CI per 10 dB L_{den} increase). 30

Figure S12: Cohort-specific results performed through interaction analyses for overweight, obesity and central obesity in relation to railway noise (OR and 95% CI per 10 dB L_{den} increase). 32

Table S1: Detailed information on the participating cohorts and outcome assessment method.

Cohort	Detailed cohort information	Key references	Funding
**DCH**	The inclusion criteria for the Danish Diet Cancer and Health (DCH) cohort were age between 50 and 64 y, residing in the greater Copenhagen or Aarhus area and without a cancer diagnosis. From 1993 to 1997, 160,725 Danes were invited to participate of whom 57,053 participants accepted the invitation and were enrolled in the study. All participants completed detailed questionnaires at enrolment and trained staff members measured height, weight, and waist circumference.	Tjonneland A, Olsen A, Boll K, et al. Study design, exposure variables, and socioeconomic determinants of participation in Diet, Cancer and Health: a population-based prospective cohort study of 57,053 men and women in Denmark. Scand J Publ Health 2007;35:432-41 DOI: 10.1080/14034940601047986	The Danish Cancer Society
**DNC**	The Danish Nurse Cohort (DNC) was initiated by sending questionnaires to the members of the Danish Nurse Organization in 1993 and 1999. Among 33,704 eligible female nurses aged 44-93 years who either worked or were retired in 1993 or 1999, 28,731 participants (85·2%) were included in the DNC. Upon enrolment, participants answered a comprehensive questionnaire on body mass index (BMI), lifestyle factors (smoking, alcohol consumption, physical activity, and dietary habits), self-reported diseases and reproductive health, and working conditions. Self-reported waist circumference is available for participants enrolled in 1999.	Hundrup, Yrsa A, Jørgensen T, Obel EB. Cohort profile: the Danish nurse cohort. Int J Epidemiol 2012; 41:1241-47. DOI: 10.1093/ije/dyr042	The Danish Council for Independent Research (DFF-4183-00353).
**MDC**	The Malmö Diet and Cancer (MDC) study is a population based prospective cohort study with a total number of 28,098 study subjects. People were enrolled into the cohort between 1991 and 1996, and eligible participants were men born between 1923 and 1945 and women born between 1923 and 1950, living in the city of Malmö. People were 44-73 years at enrolment. Swedish reading and writing skills were required. The data collection was done both using questionnaires and interviews, including data on dietary habits, socio-economics, medical history and lifestyle factors. At the visit, height and weight as well as body composition and blood pressure were measured.	Berglund G, Elmstahl S, Janzon L et al. The Malmö Diet and Cancer study. Design and feasibility. J Intern Med 1993;233:45–51. DOI: 10.1111/j.1365-2796.1993.tb00647.x Manjer J, Carlsson S, Elmstahl S et al. The Malmö Diet and Cancer Study: representativity, cancer incidence and mortality in participants and non-participants. Eur J Cancer Prev, 2001;10:489–499. DOI: 10.1097/00008469-200112000-00003	Swedish Research Council (VR) Infrastructure grant, Heart-Lung Foundation.
**PPS**	The Primary Prevention Study cohort (PPS) consists of a random third of all men in the city of Gothenburg born 1915–1925, recruited in 1970–1973 (n=7,495, participation rate 75%) to study predictors of cardiovascular disease. People were 46-55 years at enrolment. Participants were examined by health care professionals (e.g. height, weight, systolic and diastolic blood pressures and cholesterol levels) and filled out questionnaires on background data (e.g. occupation, smoking habits, physical activity, antihypertensive medication, psychological stress, prevalent diabetes mellitus and family history of coronary events).	Wilhelmsen L, Tibblin G, Werkö L. A primary preventive study in Gothenburg, Sweden. Preventive Med. 1972;1:153-60. Wilhelmsen L, Berglund G, Elmfeldt D et al. The multifactor primary prevention trial in Göteborg, Sweden. Eur Heart J. 1986;7:279-88. DOI: 10.1093/oxfordjournals.eurheartj.a062065	The Bank of Sweden Tercentenary Fund and the Swedish Medical Research Council.
**GOT-MONICA**	The GOT-MONICA included a total of 2,339 men and 2,536 women recruited as a random selection of residents in Gothenburg aged 25–64 years in 1990, and 1995. The participants filled out questionnaires on e.g.	Wilhelmsen L, Johansson S, Rosengren S et al. Risk factors for cardiovascular disease during the period 1985–1995 in Goteborg, Sweden. The GOT-	The Swedish Medical Research Council, the Swedish Heart and Lung Foundation, the Ingabritt and Arne Lundberg Research

	smoking habits, physical activity, hypertensive medication, psychological stress, marital status, and were also examined by health care professionals, for example with regard to height and weight, and blood pressures.	MONICA Project. J. Intern Med, 1997;242:199-211.<br>DOI: 10.1046/j.1365-2796.1997.00163.x	Fund, the Göteborg Medical Society and the Sahlgrenska University Hospital Funds
**SDPP[a]**	The Stockholm Diabetes Preventive Programme (SDPP) is a population-based prospective study aimed at investigating the aetiology of Type 2 diabetes and developing prevention strategies. It includes 7,949 subjects aged 35-56 years at recruitment 1992-1998. At the health examination, extensive questionnaires were completed on lifestyle factors, health status, socioeconomic characteristics, and psychosocial conditions. In addition, measurements were performed of weight, height, hip and waist circumference as well as of blood pressure and oral glucose tolerance.	Gudjonsdottir H, Tynelius P, Fors S, et al. Cohort Profile: The Stockholm Diabetes Prevention Programme (SDPP). International Journal of Epidemiology. 2022. DOI: 10.1093/ije/dyac147	Swedish Environmental Protection Agency, the Swedish Council for Health, Working Life and Social Research and the Swedish Heart-Lung Foundation. The SDPP cohort was additionally funded by the Stockholm County Council, the Swedish Research Council, the Swedish Diabetes Association and Novo Nordisk Scandinavia.
**SIXTY[a]**	The SIXTY cohort is based on a random sample of every third man and woman living in Stockholm County, who were born in 1937 and 1938. A total of 4,232 subjects were recruited 1997-1999 to investigate risk factors for cardiovascular disease. People were 59-61 years at enrolment. Measurements of anthropometric indices (BMI, WC and WHR) and blood pressure were made at recruitment and fasting blood samples were collected. In addition, a comprehensive questionnaire was completed, including information on socioeconomic, medical and lifestyle factors.	Wändell PE, Wajngot A, de Faire U, et al. Increased prevalence of diabetes among immigrants from non-European countries in 60-year- old men and women in Sweden. Diabetes Metab 2007;33:30–6.<br>DOI: 10.1016/j.diabet.2006.07.001	Swedish Environmental Protection Agency, the Swedish Council for Health, Working Life and Social Research and the Swedish Heart- Lung Foundation. The SIXTY cohort was additionally funded by the Stockholm County Council and the Swedish Research Council.
**SNAC-K[a]**	The Swedish National Study of Aging and Care in Kungsholmen (SNAC-K) was established 2001-2004 and included 3,363 residents aged 60-104 years in Kungsholmen, Stockholm. The aim was to investigate the ageing process and identify possible preventive strategies to improve health and care in elderly adults. Information was collected through social interviews and clinical examinations, including demographic and socioeconomic factors, life habits, medical and psychological status (including measurements of height and weight) as well as physical and cognitive functioning. Follow-up investigations are performed at intervals of three to six years depending on age (including measurements of height and weight).	Lagergren M, Fratiglioni L, Hallberg IR, et al. A longitudinal study integrating population, care and social services data. The Swedish National study on Aging and Care (SNAC). Aging Clin Exp Res 2004;16:158–68.<br>DOI: 10.1007/BF03324546	Swedish Environmental Protection Agency, the Swedish Council for Health, Working Life, the Swedish Research Council and Social Research and the Swedish Heart- Lung Foundation. SNAC-K was additionally funded by the Ministry of Health and Social Affairs, Sweden, Stockholm County Council and the participating Municipalities and University Departments.
**SALT[a]**	The Screening Across the Lifespan Twin Study (SALT) included a total of about 45 000 twins born 1958 and earlier from the Swedish Twin Registry who were phone interviewed 1998-2002. Those 7,043 who resided in Stockholm County at recruitment are included in the present project. People were 42-97 years at enrolment. The interview collected data on zygosity, obesity (BMI), diseases, use of medication, occupation, education and lifestyle habits. In a subgroup of around 2,500 subjects, a clinical examination was made, including blood sampling and anthropometrics as well as blood pressure measurements.	Lichtenstein P, De Faire U, Floderus M et al. The Swedish Twin Registry: a unique resource for clinical epidemiological and genetic studies. J Intern Med. 2002;252(3):184-205.<br>DOI: 10.1046/j.1365-2796.2002.01032.x<br>Lichtenstein P, Sullivan PF, Cnattingius S, et al. The Swedish Twin Registry in the third millennium: an update. Twin Res Hum Genet 2006;9(6):875–82.<br>DOI: 10.1375/183242706779462444	Swedish Environmental Protection Agency, the Swedish Council for Health, Working Life and Social Research and the Swedish Heart- Lung Foundation. The SALT cohort was additionally supported by NIH grant 575 AG-08724. The Swedish Twin Registry is managed by Karolinska Institutet and receives funding through the Swedish Research Council under the grant no 2017-00641.

**SMC**	The Swedish Mammography Cohort (SMC) is part of SIMPLER (www.simpler4health.se), a population-based prospective cohort study established between 1987-90. In the present project, we used data of 20,407 SMC women living in Uppsala County in 1997 who filled extended questionnaire including self-reported height and weight, with available information on exposure to air pollution, road traffic noise and urban greenness.	Harris H, Håkansson N, Olofsson C, Stackelberg O, Julin B, Åkesson A, Wolk A. The Swedish mammography cohort and the cohort of Swedish men: study design and characteristics of two population based longitudinal cohorts. OA Epidemiology, 2013 Oct 01;1(2):16. DOI: 10.13172/2053-079X-1-2-943	We acknowledge SIMPLER for provisioning of facilities and experimental support. SIMPLER receives funding through the Swedish Research Council under the grant no 2017-00644.
**FINRISK**	The national FINRISK study is a set of large population surveys that have been conducted to monitor non-communicable disease risk factors, health behavior and their changes in the population. Every five years during 1972-2012, a stratified random sample has been selected from the 25-74 (64 in earlier surveys) year old inhabitants in different regions of Finland. For the NordSOUND study, data from four surveys (1997, 2002, 2007, 2012) and two study areas (the cities of Helsinki/Vantaa and Turku region in Southwestern Finland) were included (n=8320). The age group 25 to 74 years is covered otherwise except that for 1997 and 2002 the age group is 25 to 64 years in the Turku region.8,320). The surveys included a self-administered questionnaire and a clinical examination with measurements of height, weight and blood pressure and blood sampling.	Borodulin K, Tolonen H, Jousilahti P et al. Cohort Profile: The National FINRISKStudy. Int J Epid, 2018;47:696-696i. DOI: 10.1093/ije/dyx239	Funding was mostly provided by the Ministry of Social Affairs and Health / Finnish Institute for Health and Welfare.<br><br>The questionnaire data used for the research was obtained from THL Biobank (study numbers BB2019_13 and THLBB2021_8). We thank all study participants for their generous participation at THL Biobank and the National FINRISK Study. Statistics Finland provided data on socioeconomic factors.

Table S2: Detailed information on estimation of road traffic, railway, and aircraft noise for the participating cohorts.

Cohort	Description	Calculation years	Key references
	**Road traffic noise estimation**		
DCH	Calculations were conducted using the Nordic prediction method implemented in SoundPLAN (version 8.0). Various input variables were used in the model, most importantly geocode and height (floor) for each address, information on travel speed, light/heavy vehicle distributions, road type, annual average daily traffic for all Danish road links (Jensen et al 2019) and 3D information on all Danish buildings. Screening effects from buildings, terrain, and noise barriers were included. All road traffic sources within 1500 m from the receivers were included. The parameters were set to allow 2 reflections.	1995, 2000, 2005, 2010, 2015	Thacher JD, Poulsen AH, Raaschou-Nielsen O, et al. High-resolution assessment of road traffic noise exposure in Denmark. Environ Res 2019; 182:109051.<br><br>Jensen SS, Plejdrup MS, Hillig K. GIS-based National Road and Traffic Database 1960-2020. Aarhus University, Danish Centre for Environment and Energy 2019; Report 151.
DNC	Same method as for DCH.		
MDC	Estimated using the Nordic Prediction Method implemented in SoundPLAN (version 8.0, SoundPLAN Nord ApS). Input variables included geocode, information on annual average daily traffic for all road links in Malmö municipality, distribution of light/heavy traffic, signposted travel speed and road type and polygons for all buildings in Malmö. All road traffic sources within 1000 m from the receivers were included. Traffic data were retrieved from a regional emission database (Rittner et al. 2020). The screening effects from buildings were included and ground softness considered. The parameters in the models were set to allow 2 reflections and receivers placed at 2 m height. Estimation of road traffic noise exposure between years with models was based on the model closest in time or the year of major changes in infrastructure, i.e. the model from 1990 was used for residential coordinates 1985-1999, the model from 2000 used for coordinates 2000-2005 and the model from 2010 for coordinates between 2006 and 2016.	1990, 2000, 2010	Bendtsen, H., 1999. The nordic prediction method for road traffic noise. Sci. Total Environ. 235, 331–338. https://doi.org/10.1016/S0048-9697(99)00216-8.<br><br>Rittner R, Gustafsson S, Spanne M, Malmqvist E. Particle concentrations, dispersion modelling and evaluation in southern Sweden, SN Applied Sciences 2020;2:1013.
PPS, GOT-MONICA	Yearly average road traffic flows, speed and percentage of heavy vehicles were obtained from the environmental office of the municipality of Gothenburg and the traffic office of the municipality of Mölndal. The traffic flow estimations were based on measurements for all major and medium links but used a standard default flow for very small streets. Terrain data and building footprints were obtained from Lantmäteriet and road links from the Swedish National Traffic Administration. Noise barriers of at least 2 m height and 100 m length were also included, and earth berms were included in the terrain model. To	1975-2010, yearly	Ögren M, Barregard L. Road traffic noise exposure in Gothenburg1975-2010. PLoS One 2016;11:e015532.

Cohort	Description	Calculation years	Key references
	save calculation time and reduce demands on detailed input data a simplified methodology was used for multiple reflections in dense urban areas.		
SDPP, SIXTY, SNAC-K, SALT	To assess long-term individual transportation noise exposure a noise database for Stockholm County was developed representing the period from 1990 and onwards, with detailed estimation every fifth year. The database includes 3D terrain data as well as information on ground surface, road net, daily traffic flows (≥1000 vehicles/day), speed limits and percentage of heavy vehicles. To calculate noise levels for road traffic a modification of the Nordic prediction method was used, where possible reflection and shielding were taken into account by a Ground Space Index based on building density. The methodology has been further developed from the one described by Ögren and Barregard (2016), which was validated against the full Nordic prediction method modelled with SoundPlan and showed coherent estimates.	1990, 1995, 2000, 2005, 2010, 2015	Ögren M, Barregard L. Road traffic noise exposure in Gothenburg1975-2010. PLoS One 2016;11:e015532.
SMC	Exposure to road traffic noise was assessed using the Nordic Prediction Method (Bendtsen 1999). Input data included ground surface (assuming flat terrain), road net and traffic flows (simulated and calibrated against historical measurements) on both state owned and municipal roads, diurnal distributions, percentage heavy vehicles, speed, and buildings. The exposure was calculated as free-field levels at the façade of the buildings at 2 m height. Within urban areas (primarily within the city of Uppsala), the calculations were performed with second-order reflections, whereas in more rural areas, first-order of reflections were used. The search radii were set to 1,000 m within Uppsala and to 1,500 m in more rural areas. The estimated sound parameter, L_{den}, was calculated for each address every fifth year from 1990 to 2015. To calculate yearly averages, linear interpolation was applied.	1990, 1995, 2000, 2005, 2010, 2015	Bendtsen, H., 1999. The nordic prediction method for road traffic noise. Sci. Total Environ. 235, 331–338. https://doi.org/10.1016/S0048-9697(99)00216-8.
FINRISK	Façade noise levels from road-traffic were calculated by consulting companies in accordance with the EU Environmental Noise Directive 2002/49/EC50 using input data for the year 2011. The Nordic Prediction Method was used for major highways, main streets, and collector streets within Helsinki, Vantaa, and Turku. Input variables included terrain elevation data, traffic flows on the road network, speed limits and percentage of heavy vehicles, locations and heights of noise barriers. In addition, bridges, road profiles as well as acoustic hardness of terrain or water surfaces were specified. All road traffic sources within 2000 m from the receivers were included. Counting height was 4 m. The parameter setting were set to allow 1 reflection, but not from the facade in question. No	2011	Sito Oy. 2012. Pääkaupunkiseudun ympäristömeludirektiivin mukainen meluselvitys. https://docplayer.fi/6614750-Paakaupunkiseudun-ymparistomeludirektiivin-mukainen-meluselvitys-yhdistelmaraportti.html<br><br>Pöyry Finland Oy. 2012. Ympäristömeludirektiivin mukainen ympäristömeluselvitys Turussa.<br><br>https://www.turku.fi/sites/default/files/atoms/files/

Cohort	Description	Calculation years	Key references
	weather corrections were used. The highest L_{den} on façade points within 20 m of residential address coordinates was assigned as the noise exposure to that dwelling.		/ymparistomeluselvitys_turussa_loppuraportti_1.6.2012.pdf
	**Railway noise estimation**		
DCH	Calculations were conducted using the Nord 2000 method implemented in SoundPLAN (version 8.0). Various input variables were used in the model, most importantly geocode and height (floor) for each address, information on train speed and type, annual average trains for the day, evening and night and 3D information on all Danish buildings within 1000 m of the railway. Screening effects from buildings, terrain, and noise barriers were included. All rail traffic sources within 1500 m from the receivers were included. The parameters were set to allow 2 reflections.	1997, 2012	B Plovsing, J Kragh (2006) Nord2000. Validation of the propagation model. Delta Acoustics Ringheim, M. The new Nordic prediction method for railway noise. Journal of sound and vibration 193.1 (1996): 277-282 and Vibration Report vol. AV 1117/06
DNC	Same method as for DCH.		
MDC	Estimated using the Nordic Prediction Method implemented in SoundPLAN (version 8.0, SoundPLAN Nord ApS). Input variables included geocode, information on annual average daily traffic and speed for all railways, train types and polygons for all buildings in Malmö. All railway traffic sources within 1000 m from the receivers were included. Screening effects from buildings were included and ground softness was considered. The parameters in the models were set to allow 2 reflections and receivers placed at 2 m height. Estimation of railway noise exposure between years with models was based on the model closest in time or the year of major changes in infrastructure. Level day-evening-night was estimated from the equivalent level using an adjustment of 6 dB.	1990, 2000, 2010	Ringheim, M. The new Nordic prediction method for railway noise. Journal of sound and vibration 193.1 (1996): 277-282
PPS, GOT-MONICA	Yearly traffic counts (separate for day, evening and night), train speed and composition of different train types were obtained from the environmental office of the municipality of Gothenburg and the traffic office of the municipality of Mölndal for trams. For standard rail traffic, the same information was obtained from the Swedish traffic administration. Before 1997 regional and national rail transport statistics and published timetables were used to estimate traffic counts. Noise barriers of at least 2 m height and 100 m length were also included, as well as earth berms in the terrain model. To save calculation time and reduce demands on detailed input data a simplified methodology was used for multiple reflections in dense urban areas. All receivers were assumed to be at 2 m height above ground at the address coordinate.	1975-2010, yearly	Ögren M, Barregard L. Road traffic noise exposure in Gothenburg1975-2010. PLoS One 2016;11:e015532.

Cohort	Description	Calculation years	Key references
SDPP, SIXTY, SNAC-K, SALT	Estimations were based on a noise database for Stockholm County which represented the period from 2010 and retrospectively until 1997, with detailed annual estimates. The database includes 3D terrain data as well as information on ground surface, railway net, speed limits and annual average daily train flows separately for light and heavy trains. To calculate noise levels for railway traffic a modification of the Nordic prediction method was used, where possible reflection and shielding were taken into account by a Ground Space Index based on building density. The methodology has been adopted and further developed from the one for road traffic noise described by Ögren and Barregard (2016), which was validated against the full Nordic prediction method modelled with SoundPlan and showed coherent estimates. Level day-evening-night was estimated from the equivalent level using an adjustment of 6 dB.	1997-2010	Ringheim, M. The new Nordic prediction method for railway noise. Journal of sound and vibration 193.1 (1996): 277-282<br><br>Ögren M, Barregard L. Road traffic noise exposure in Gothenburg1975-2010. PLoS One 2016;11:e015532.
SMC	Railway noise was not assessed.		
FINRISK	Façade noise levels from major railways (> 30.000 train passages a year) were calculated by consulting companies in accordance with the EU Environmental Noise Directive 2002/49/EC50 using input data for the year 2011 for trains, and year 2016 for trams and underground. In the 2012 assessment, the Nordic Prediction Method was used for train traffic in Helsinki, Vantaa and Turku, while the Common Noise Assessment Methods in Europe (CNOSSOS-EU) was used in the 2017 assessment for trams and underground in Helsinki. Input variables included traffic data (number of passages, types and lengths of trains, speed), terrain elevation data, buildings, and locations and heights of noise barriers. In addition, acoustic hardness of terrain or water surfaces were specified. All rail traffic sources within 2000 m from the receivers were included. Counting height was 4 m. The parameter settings were set to allow 1 reflection, but not from the facade in question. The highest L_{den} on façade points within 20 m of residential address coordinates was assigned as the noise exposure to that dwelling.		The Assessment of Road and Railway Traffic Noise of Finnish Transport Agency 2012. Finnish Transport Agency, Transport System. Helsinki 2012. 34 pages. ISBN 978-952-255-158-0.<br><br>Road-traffic and Rail-traffic Noise Assessment of the Finnish Transport Agency 2017. Finnish Transport Agency, Engineering and Environment. Helsinki 2017. 25 pages and 1 appendix. ISBN 978-952-317-507-5.
	**Aircraft noise estimation**		
DCH	The noise impact from all Danish airports and airfields was calculated by local authorities using the programs DANSIM (Danish Airport Noise Simulation Model) and INM3 (Integrated Noise Model), which meet joint Nordic criteria for air traffic noise calculations.	1986-2009	Liasjø, K. H., & Granøien, I. L. N. Sammenligning av flystøyberegningsprogrammerne INM-2/6, INM-3/9, INM-3/10, DANSIM og NOISEMAP (Beregninger og målinger vedr. Fomebu) [in Norwegian]. 1993.

Cohort	Description	Calculation years	Key references
DNC	Same method as for DCH.		
SDPP, SIXTY, SNAC-K, SALT	Noise contours for years the 1995 to 2013 for both Stockholm airports have been estimated by Swedavia in accordance with Swedish and international methods using the aircraft noise modelling software INM. Although local conditions such as arrival and departure procedures have been taken into account, it is particularly difficult to calculate accurate levels as low as 45 dB (A). Results show that the changes of noise footprints throughout the years are principally due to changes in aircraft fleet or flight paths.	1995, 2000, 2005, 2010, 2013	Swedavia technical report D 2015-003915<br><br>Integrated noise model (INM) version 7.0 technical manual, Report Number: FAA-AEE-08-01 https://rosap.ntl.bts.gov/view/dot/12188
SMC	Not assessed.		
FINRISK	In FINRISK, aircraft noise for 2011 was modelled in 5 dB categories >50 dB according to the European Civil Aviation Conference report.	2011	European Civil Aviation Conference. Report on Standard Method of Computing<br><br>Noise Contours around Civil Airports. Volume 1: Applications Guide In. 3rd ed. Neuilly-sur-Seine Cédex: ECAC-CEAC; 2005.

Table S3: Detailed information on estimation of air pollution for the participating cohorts.

Cohort	Air pollution estimation	Key references
DCH	In the DCH cohort, we used the DEHM-UBM-AirGIS modelling system to calculate $PM_{2.5}$ and NO_2 at all Danish addresses for the years 2000, 2010 and 2015, which was then extrapolated to yearly means for each address, based on changes in yearly urban background levels. This multi-scale dispersion modelling system calculates air pollutants at each address as the sum of a) $PM_{2.5}$/NO_2 from the nearest street, calculated based on traffic, car fleet emission factors, streets and building geometry, and meteorology; b) urban background, calculated based on city dimensions, emission density, and heights of buildings; and c) regional background, calculated based on all emissions in the northern hemisphere.	Khan J, Kakosimos K, Raaschou-Nielsen O, et al. Development and performance evaluation of new AirGIS – A GIS based air pollution and human exposure modelling system. Atmos Environ 2019;198:102-121.<br><br>Ketzel M, Berkowicz R, Hvidberg M, Jensen SS and Raaschou-Nielsen O. Evaluation of AirGIS - a GIS-based air pollution and human exposure modelling system. Int J Environ Pollution 2011;47:226–238.<br><br>Brandt, J, Christensen JH, Frohn LM and Berkowicz R, Air pollution forecasting from regional to urban street scale – implementation and validation for two cities in Denmark. Physics and Chemistry of the Earth, 2003.;28:335-344.
DNC	Same method as for DCH.	
MDC	Air pollutants ($PM_{2.5}$, and nitrogen oxides converted to NO_2) were modelled using EnviMan (Opsis AB, Sweden) by the Environmental Department, City of Malmö, using a Gaussian dispersion model (AERMOD) combined with an emission database for the county of Scania in Sweden. The 18 × 18 km modelling area covered the city of Malmö and the closest surroundings. Separate emission databases were compiled for 1992, 2000 and 2011 using existing local and regional bottom-up inventories provided by the municipality, and then supplemented to be consistent for the whole area and time-period. Yearly mean concentrations were stored as grids with a spatial resolution of 50 m × 50 m. The years in between the modelled years were interpolated linearly with adjustment for year-to-year variations in the local meteorology using a ventilation factor estimated from calculations over the whole time-period, and exposure for the years 1990 and 1991 extrapolated. Exposure data was combined with geocoded addresses to assign each participant annual residential exposures.	Hasslöf H, Molnár P, Andersson EM, et al. Long-term exposure to air pollution and atherosclerosis in the carotid arteries in the Malmö Diet and Cancer Cohort. Environ Res 2020:110095<br><br>Xu Y, Andersson, EM, Carlsen HK et al. Associations between long-term exposure to low-level air pollution and risk of chronic kidney disease – findings from the Malmö Diet and Cancer cohort. Env Int 2022:107085
PPS	The exposure assessment was performed similarly for Gothenburg and Stockholm as part of the Swedish Clean air and Climate Research program (SCAC). For Gothenburg, high-resolution dispersion modelling of source-specific nitrogen oxides converted to NO_2 was performed over an area of 93 × 112 km for the years 1990, 2000 and 2011. Emission inventories were compiled using local and regional bottom-up inventories provided by the municipality and supplemented to be consistent for the whole time-period. Intervening years were interpolated so that each participant could be assigned annual residential air pollutant exposures.	Segersson D, Eneroth K, Gidhagen L et al. Health impact of PM_{10}, $PM_{2.5}$ and black carbon exposure due to different source sectors in Stockholm, Gothenburg and Umea, Sweden. Int J Environ Res Public Health, 2017;14:E742.<br><br>Stockfelt L, Andersson EM, Molnár P, et al. Long-term effects of total and source-specific particulate air pollution on incident

		cardiovascular disease in Gothenburg, Sweden. Environ Res 2017;158:61-71.
GOT-MONICA	Same method as for PPS.	
SDPP, SIXTY, SNAC-K, SALT	In the Stockholm County cohorts, a high-resolution Gaussian dispersion model was used to estimate individual residential levels of $PM_{2.5}$ and NO_x/NO_2 using local emission inventories every fifth year from 1990 and onwards. The emission inventory contains detailed information on local emissions from road and ferry traffic, industrial areas and households. Meteorological input to the modelling includes measurements of wind velocity and direction, solar radiation and temperature. Further, a street canyon contribution is added for addresses in the most polluted street segments of the inner city of Stockholm with multi-storey houses on both sides. Annual averaged long-range contributions were added to the locally modelled concentrations based on continuous measurements at regional background monitoring stations.	Segersson D, Eneroth K, Gidhagen L, et al. Health impact of PM_{10}, $PM_{2.5}$ and black carbon exposure due to different source sectors in Stockholm, Gothenburg and Umea, Sweden. Int J Environ Res Public Health 2017;14:E742.<br><br>Ljungman PLS, Andersson N, Stockfelt L, et al. Long-term exposure to particulate air pollution, black carbon, and their source components in relation to ischemic heart disease and stroke. Environ Health Perspect 2019;127:107012
SMC	Exposure to air pollution was assessed by SLB Analys (https://www.slb.nu/slbanalys/) using a dispersion modelling (Segersson et al. 2017). Sources considered were emissions from road traffic (including street canyon effect where applicable), boilers and energy plants, individual heating with solid fuel (wood) and oil, shipping, and long-range transport. Yearly average concentrations of $PM_{2.5}$, PM_{10} and NO_2 for all sources combined were calculated for all addresses of our study participants.	Segersson D, Eneroth K, Gidhagen L et al. Health impact of PM10, PM2.5 and black carbon exposure due to different source sectors in Stockholm, Gothenburg and Umea, Sweden. Int J Environ Res Public Health, 2017;14:E742.
FINRISK	Estimates of $PM_{2.5}$ and NO_2 concentrations were based on dispersion modelling using models developed in the Finnish Meteorological Institute. The calculations included emissions from energy production, industry, ship traffic and road traffic. The average measured background was added to the concentrations. Emissions used in the calculations represented the situation in Turku Region in 2007 and 2014 in the Helsinki Capital Region. In the dispersion models, the distances between the receptor points varied from 25 m near the roads to 500 m in rural areas. In the present study, the modelled annual average concentration at the nearest outdoor receptor point was used as a proxy of home outdoor concentration.	Salmi, J., Lappi, S., Rasila, T., Lovén, K. ja Hannuniemi, H. 2009. Turun seudun päästöjen leviämismalliselvitys. Finnish Meteorological Institute. Helsinki. http://expo.fmi.fi/aqes/public/Turun_seudun_leviamismallilaskelmat_2010.pdf<br><br>Hannuniemi, H., Salmi, J., Rasila, T., Wemberg, A., Komppula, B., Lovén, K. ja Pietarila, H. 2016. Pääkaupunkiseudun päästöjen leviämismalliselvitys. Finnish Meteorological Institute. Helsinki. http://expo.fmi.fi/aqes/public/PKS_Ilmanlaaturaportti_01.06.2016.pdf

Table S4: Spearman correlation coefficients between road traffic noise, railway noise, $PM_{2.5}$ and NO_2.

	**Road traffic noise**	**Railway noise**	**$PM_{2.5}$**	**NO_2**
**Road traffic noise**	—			
**Railway noise**	0.15	—		
**$PM_{2.5}$[a]**	0.36	-0.06	—	
**NO_2[b]**	0.56	0.14	0.41	—

Note: Includes 137,657 participants from the cohorts DCH, DNC, MDC, GOT-Monica, SDPP, SIXTY, SNAC-K, SALT and FINRISK.

[a] $PM_{2.5}$, particulate matter with an aerodynamic diameter of ≤2,5 μm (fine particulate matter).

[b] NO_2, nitrogen dioxide.

Table S5: Pooled and cohort-specific results for aircraft noise.

Obesity marker		POOLED	DCH	DNC	SDPP	SIXTY	SNAC-K	SALT	FINRISK
Linear regression, b (95% CI) per 10 dB L_{den}									
BMI (kg/m²)	<40 dB	0.00 (ref)	0.00 (ref)	0.00 (ref)	0.00 (ref)	0.00 (ref)	0.00 (ref)	0.00 (ref)	0.00 (ref)
	40–49 dB	0.01 (-0.13;0.15)	-0.46 (-0.87;-0.05)	-0.09 (-0.77;0.59)	0.11 (-0.21;0.43)	-0.08 (-0.47;0.30)	0.24 (-0.21;0.69)	0.06 (-0.19;0.30)	-0.25 (-1.48;0.98)
	≥50 dB	-0.15 (-0.3;0.00)	-0.13 (-0.52;0.25)	-0.16 (-0.63;0.31)	-0.01 (-0.26;0.24)	0.5 (-0.26;1.26)	-0.25 (-0.80;0.29)	-0.13 (-0.60;0.35)	0.19 (-0.32;0.69)
Waist circumference (cm)	<40 dB	0.00 (ref)	0.00 (ref)	0.00 (ref)	0.00 (ref)	0.00 (ref)	—	—	0.00 (ref)
	40–49 dB	-0.5 (-1.01;0.04)	-1.19 (-2.27;-0.10)	-5.48 (-9.49;-1.46)	-0.47 (-1.24;0.29)	-0.26 (-1.29;0.78)	—	—	-0.18 (-3.37;3.01)
	≥50 dB	-0.2 (-0.68;0.28)	-0.32 (-1.34;0.70)	-0.49 (-3.20;2.23)	-0.21 (-0.8;0.38)	0.56 (-1.49;2.61)	—	—	0.36 (-0.94;1.67)
Logistic regression, OR (95% CI)									
Overweight[a]	<40 dB	1.00 (ref)	1.00 (ref)	1.00 (ref)	1.00 (ref)	1.00 (ref)	1.00 (ref)	1.00 (ref)	1.00 (ref)
	40–49 dB	1.03 (0.96;1.12)	0.76 (0.61;0.94)	1.19 (0.78;1.82)	1.13 (0.95;1.33)	1.16 (0.95;1.41)	0.98 (0.78;1.23)	1.06 (0.91;1.24)	0.96 (0.53;1.74)
	≥50 dB	0.92 (0.85;1.00)	0.85 (0.69;1.04)	0.87 (0.64;1.19)	0.99 (0.87;1.13)	1.22 (0.82;1.82)	0.81 (0.61;1.06)	0.94 (0.69;1.26)	1.16 (0.90;1.49)
Obesity[b]	<40 dB	1.00 (ref)	1.00 (ref)	1.00 (ref)	1.00 (ref)	1.00 (ref)	1.00 (ref)	1.00 (ref)	1.00 (ref)
	40–49 dB	1.06 (0.93;1.20)	0.90 (0.64;1.25)	0.55 (0.17;1.77)	1.2 (0.93;1.56)	1.04 (0.79;1.37)	1.32 (0.91;1.92)	0.97 (0.71;1.33)	0.41 (0.12;1.42)
	≥50 dB	0.86 (0.75;0.98)	0.85 (0.62;1.17)	0.51 (0.23;1.17)	0.94 (0.77;1.16)	1.10 (0.62;1.92)	0.96 (0.61;1.52)	0.87 (0.46;1.65)	1.13 (0.80;1.59)
Central obesity[c]	<40 dB	1.00 (ref)	1.00 (ref)	1.00 (ref)	1.00 (ref)	1.00 (ref)	—	—	1.00 (ref)
	40–49 dB	0.95 (0.85;1.08)	0.80 (0.62;1.04)	0.81 (0.31;2.10)	1.03 (0.83;1.27)	0.93 (0.76;1.14)	—	—	1.11 (0.55;2.21)
	≥50 dB	0.90 (0.80;1.01)	0.94 (0.74;1.19)	1.12 (0.60;2.07)	0.90 (0.75;1.06)	0.89 (0.60;1.32)	—	—	0.99 (0.76;1.28)

Note: —, Not applicable; BMI, Body Mass Index; DCH, Diet, Cancer and Health cohort; DNC, Danish Nurses Cohort; GOT-MONICA, Multinational Monitoring of Trends and Determinants in Cardiovascular Disease cohort (Gothenburg); MDC, Malmö Diet and Cancer Study; PPS, Primary Prevention Study cohort; SALT, Stockholm Screening Across the Lifespan Twin Study; SDPP, Stockholm Diabetes Prevention Programme; Sixty, the Stockholm Cohort of 60-year-olds; SMC, The Swedish Mammography Cohort; SNAC-K, Swedish National Study of Aging and Care in Kungsholmen.
All estimates are expressed as per 10 dB Lden increase.
Adjusted for cohort, sex, age, recruitment year, educational level, marital status, area income, smoking status and physical activity.
[a]Overweight: Body Mass Index ≥25 kg/m².
[b]Obesity: Body Mass Index ≥30 kg/m².
[c]Central obesity: Women: ≥88 cm, men: ≥102 cm.

Table S6: Sensitivity analyses for road traffic noise in relation to overweight, obesity and central obesity (OR and 95% CI per 10 dB L_{den} increase).

	Overweight[a]		Obesity[b]		Central obesity[c]	
	N	OR (95% CI)	N	OR (95% CI)	N	OR (95% CI)
Main analysis (based on Model 2[d]), including observations with complete smoking intensity data only.	122,208	1.04 (1.02;1.05)	76,552	1.07 (1.04;1.10)	103,714	1.05 (1.03;1.07)
Additionally adjusted for continuous smoking intensity	122,208	1.03 (1.02;1.05)	76,552	1.07 (1.04;1.09)	103,714	1.05 (1.03;1.07)
Main analysis (based on Model 2[d]), including observations with complete $PM_{2.5}$ data only.	154,969	1.03 (1.01;1.04)	98,090	1.06 (1.04;1.08)	126,967	1.03 (1.01;1.05)
Additionally adjusted for continuous $PM_{2.5}$	154,969	1.03 (1.01;1.04)	98,090	1.06 (1.03;1.09)	126,967	1.02 (1.00;1.04)
Main analysis (based on Model 2[d]), including observations with complete NO_2 data only.	159,993	1.02 (1.01;1.04)	100,844	1.06 (1.04;1.08)	126,966	1.03 (1.01;1.05)
Additionally adjusted for continuous NO_2	159,993	1.04 (1.02;1.05)	100,844	1.05 (1.02;1.08)	126,966	1.02 (1.00;1.04)
Main analysis (based on Model 2[d]), including observations with complete railway and aircraft noise data only.	109,741	1.03 (1.02;1.05)	69,842	1.07 (1.04;1.10)	81,726	1.04 (1.02;1.06)
Additionally adjusted for railway noise	109,741	1.03 (1.02;1.05)	69,842	1.06 (1.04;1.09)	81,726	1.04 (1.02;1.06)
Additionally adjusted for aircraft noise	109,741	1.03 (1.02;1.05)	69,842	1.07 (1.04;1.10)	81,726	1.04 (1.02;1.06)
Main analysis (based on Model 2[d]).	162,639	1.03 (1.01;1.04)	102,823	1.06 (1.03;1.08)	127,040	1.03 (1.01;1.05)
Measured obesity markers[e]	112,606	1.05 (1.03;1.07)	66,578	1.09 (1.06;1.12)	104,840	1.06 (1.04;1.08)
Self-reported obesity markers[f]	50,033	0.98 (0.96;1.00)	36,245	0.98 (0.94;1.03)	22,200	0.92 (0.89;0.96)
Excluding DCH	107,937	0.99 (0.97;1.00)	70,879	0.99 (0.96;1.02)	72,344	0.99 (0.96;1.01)
Excluding MDC	134,815	1.02 (1.00;1.03)	86,070	1.05 (1.02;1.07)	99,229	1.01 (0.99;1.03)
Excluding DNC	136,362	1.03 (1.01;1.04)	82,552	1.06 (1.03;1.08)	119,792	1.03 (1.01;1.05)

[a] Overweight: Body Mass Index ≥25 kg/m² vs Body Mass Index <25 kg/m² as reference category.
[b] Obesity: Body Mass Index ≥30 kg/m² vs Body Mass Index <25 kg/m² as reference category.
[c] Central obesity: Women: ≥88 cm, men: ≥102 cm.
[d] Model 2, main model, adjusted for age, sex, recruitment year, cohort, educational level, marital status, smoking status, physical activity and area level income.
[e] Cohorts: DCH, GOT-Monica, PPS, MDC, SDPP, SNAC-K, SIXTY, and FINRISK.
[f] Cohorts: DNC, SALT, and SMC.

Table S7: Sensitivity analyses for railway noise in relation to overweight, obesity and central obesity (OR and 95% CI per 10 dB L_{den} increase).

	Overweight[a]		Obesity[b]		Central obesity[c]	
	N	OR (95% CI)	N	OR (95% CI)	N	OR (95% CI)
Main analysis (based on Model 2[d]), including observations with complete smoking intensity data only.	115,336	1.05 (1.03;1.07)	71,717	1.08 (1.05;1.11)	97,937	1.06 (1.04;1.09)
Additionally adjusted for continuous smoking intensity	115,336	1.05 (1.03;1.07)	71,717	1.08 (1.05;1.11)	97,937	1.06 (1.04;1.09)
Main analysis (based on Model 2[d]), including observations with complete $PM_{2.5}$ data only.	137,617	1.04 (1.02;1.06)	86,374	1.06 (1.03;1.09)	112,035	1.06 (1.04;1.08)
Additionally adjusted for continuous $PM_{2.5}$	137,617	1.04 (1.02;1.05)	86,374	1.06 (1.03;1.09)	112,035	1.06 (1.03;1.08)
Main analysis (based on Model 2[d]), including observations with complete NO_2 data only	142,642	1.04 (1.01;1.05)	89,129	1.06 (1.03;1.09)	112,035	1.06 (1.04;1.08)
Additionally adjusted for continuous NO_2	142,642	1.03 (1.02;1.05)	89,129	1.05 (1.03;1.08)	112,035	1.05 (1.03;1.07)
Main analysis (based on Model 2[d]), including observations with complete railway and aircraft noise data only.	109,741	1.02 (1.00;1.04)	69,842	1.02 (0.99;1.06)	81,726	1.04 (1.01;1.06)
Additionally adjusted for road traffic noise	109,741	1.02 (1.00;1.04)	69,842	1.02 (0.99;1.05)	81,726	1.03 (1.01;1.06)
Additionally adjusted for aircraft noise	109,741	1.02 (1.00;1.04)	69,842	1.02 (0.99;1.05)	81,726	1.04 (1.01;1.06)
Main analysis (based on Model 2[d]).	145,281	1.03 (1.02;1.05)	91,103	1.06 (1.03;1.09)	112,103	1.06 (1.04;1.08)
Measured obesity markers[e]	112,599	1.04 (1.02;1.06)	66,572	1.08 (1.05;1.11)	104,833	1.07 (1.04;1.09)
Self-reported obesity markers[f]	32,682	1.00 (0.96;1.04)	24,531	0.94 (0.86;1.01)	7,270	0.95 (0.86;1.04)
Excluding DCH	90,579	1.02 (1.00;1.05)	59,159	1.04 (1.00;1.08)	57,407	1.05 (1.01;1.08)
Excluding MDC	117,457	1.02 (1.00;1.04)	74,350	1.02 (0.99;1.05)	84,292	1.04 (1.01;1.06)
Excluding DNC	118,994	1.04 (1.02; 1.06)	70,836	1.08 (1.05; 1.11)	104,860	1.06 (1.04;1.09)

[a] Overweight: Body Mass Index ≥25 kg/m² vs Body Mass Index <25 kg/m² as reference category.
[b] Obesity: Body Mass Index ≥30 kg/m² vs Body Mass Index <25 kg/m² as reference category.
[c] Central obesity: Women: ≥88 cm, men: ≥102 cm.
[d] Model 2, main model, adjusted for age, sex, recruitment year, cohort, educational level, marital status, smoking status, physical activity and area level income.
[e] Cohorts: DCH, GOT-Monica, PPS, MDC, SDPP, SNAC-K, SIXTY, and FINRISK.
[f] Cohorts: DNC, SALT, and SMC.

Figure S1: Histograms of road traffic, railway, and aircraft noise exposure for the pooled cohort (displaying levels of ≥40 dB L_{den} only).

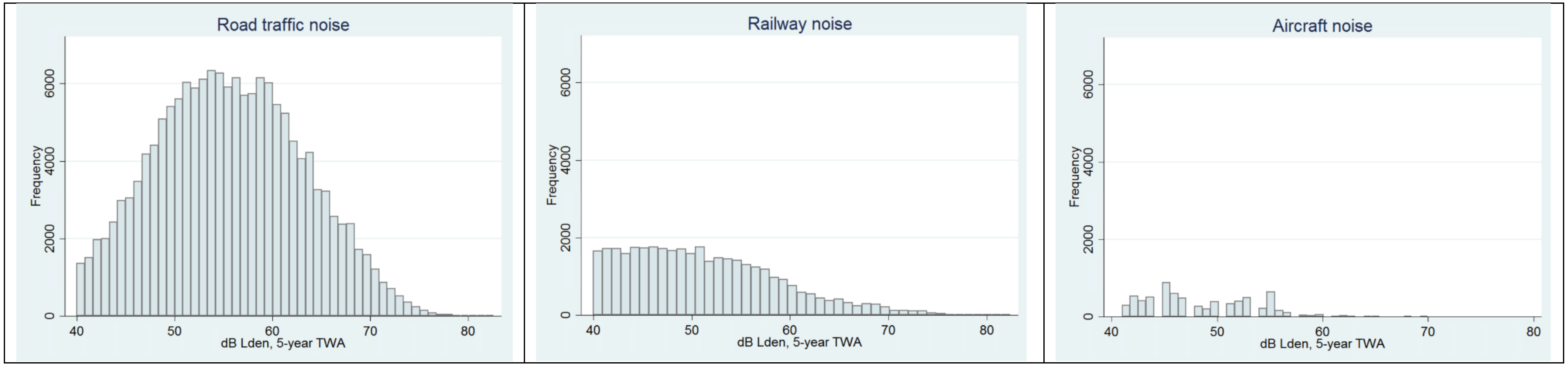

Figure S2: Cohort-specific histograms of exposure to road traffic noise (displaying levels of ≥40 dB L_{den} only).

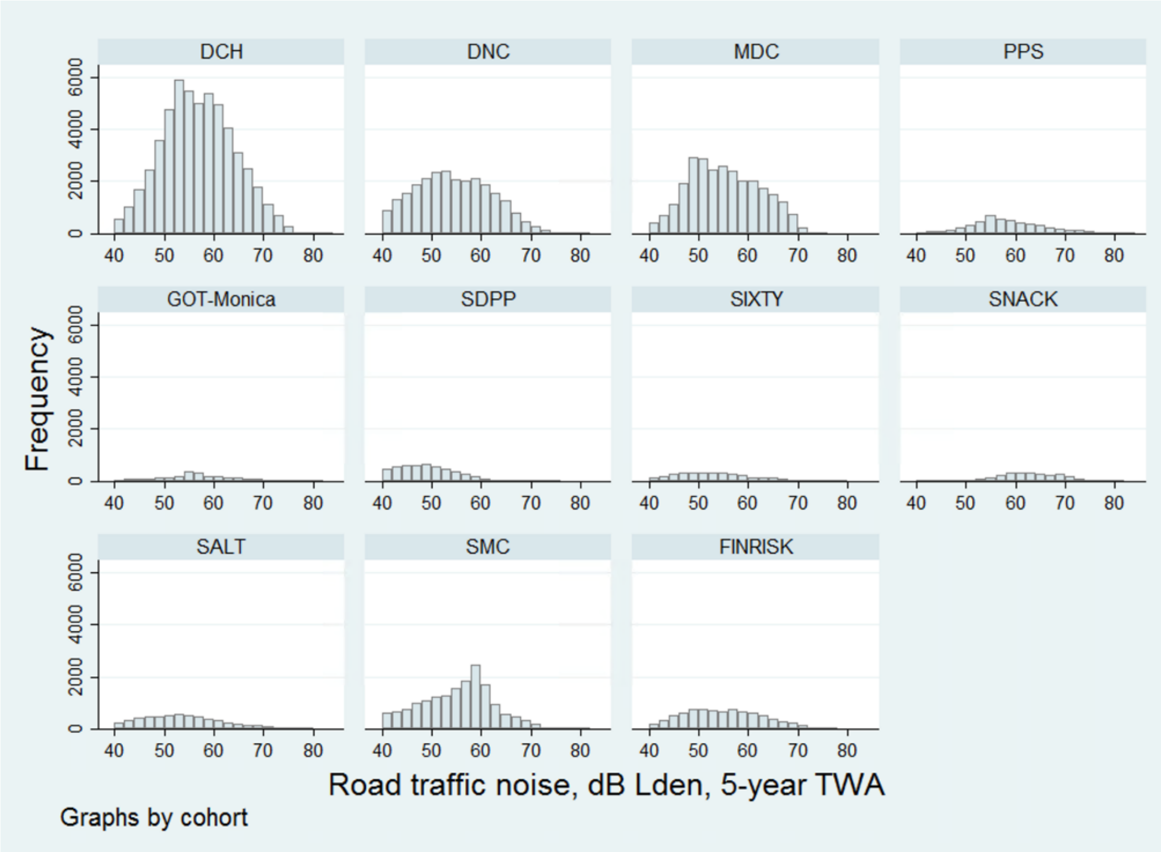

Figure S3: Cohort-specific histograms of exposure to railway noise (displaying levels of ≥40 dB L_{den} only).

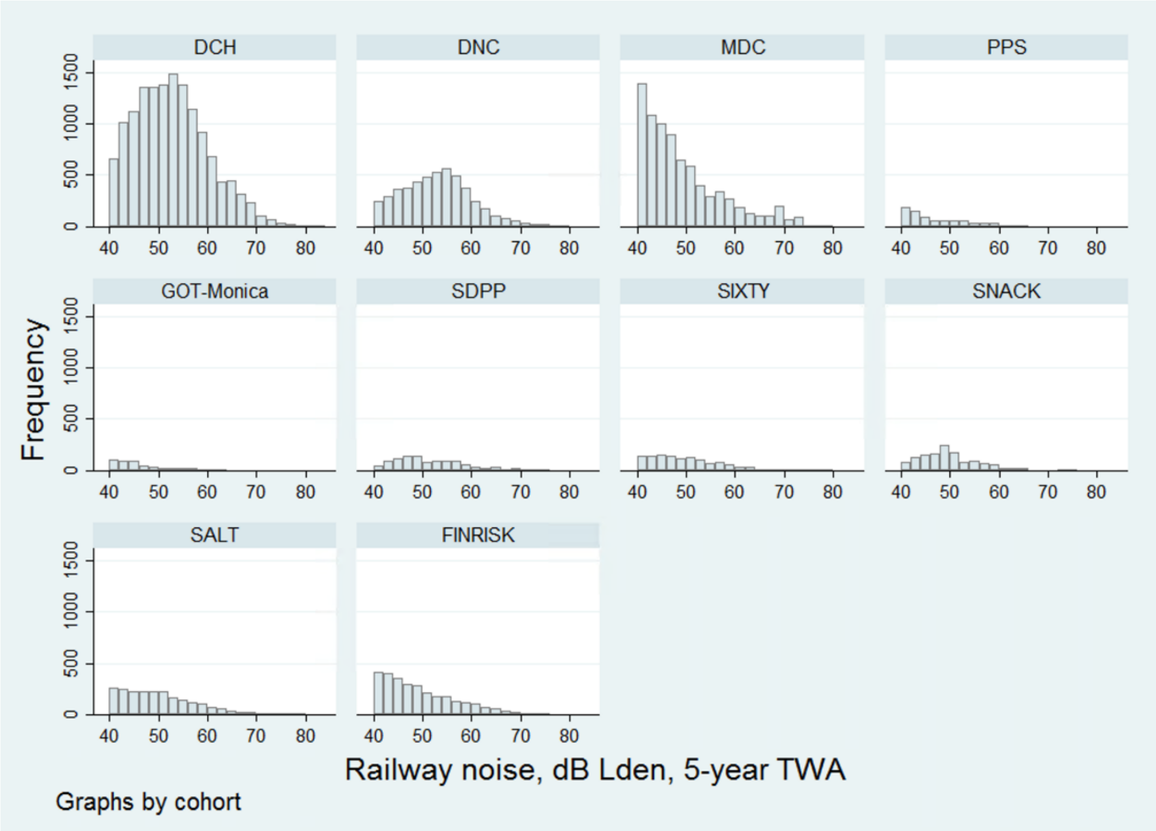

Figure S4: Cohort-specific histograms of exposure to aircraft noise (displaying levels of ≥40 dB L_{den} only). Data only available in 5 dB for DCH, DNC and FINRISK.

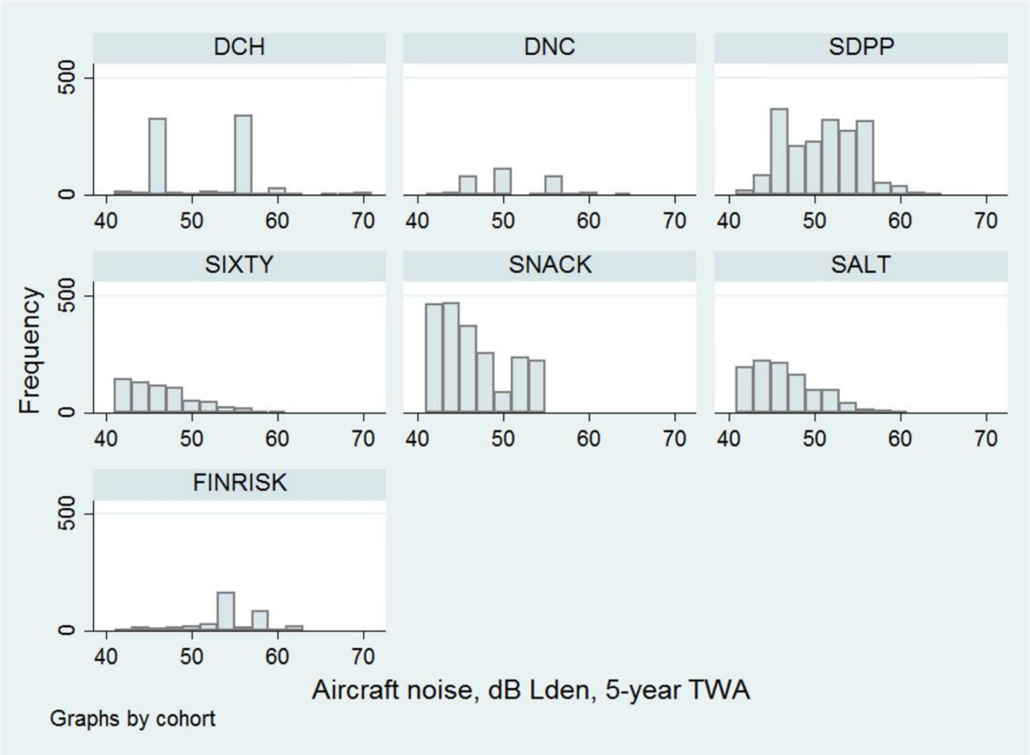

Figure S5: Histograms of air pollution exposure ($PM_{2.5}$, NO_2) for the pooled sample.

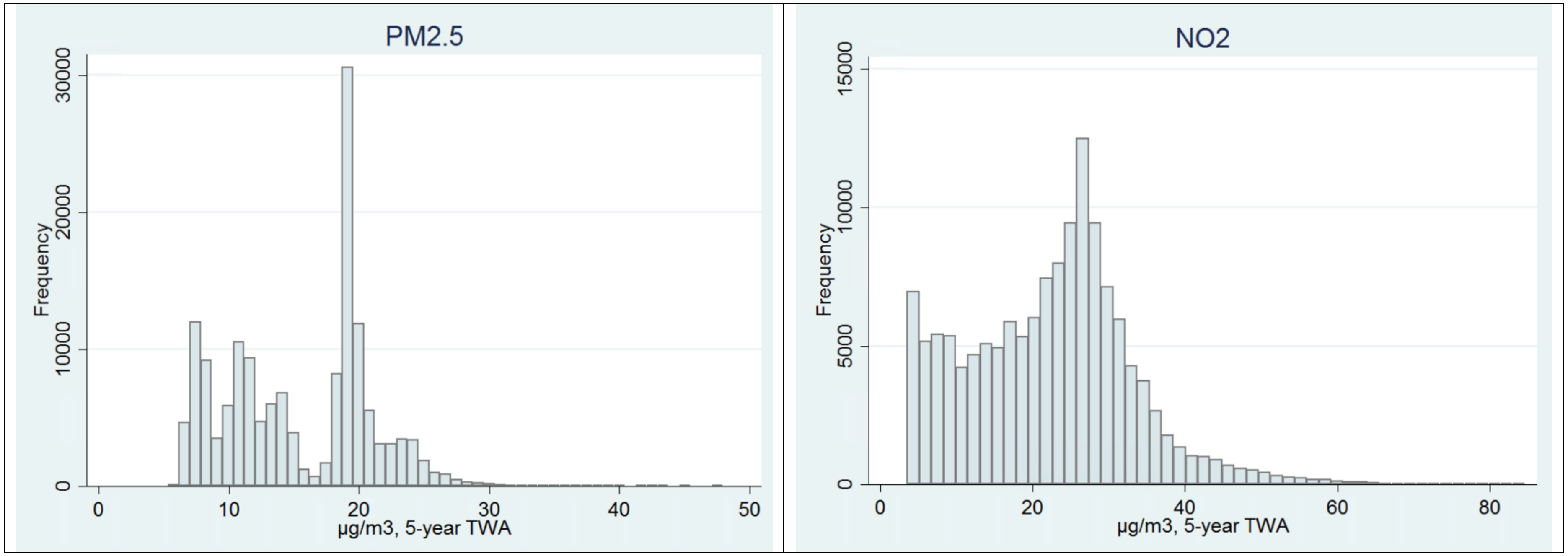

Figure S6: Cohort-specific histograms of exposure to $PM_{2.5}$.

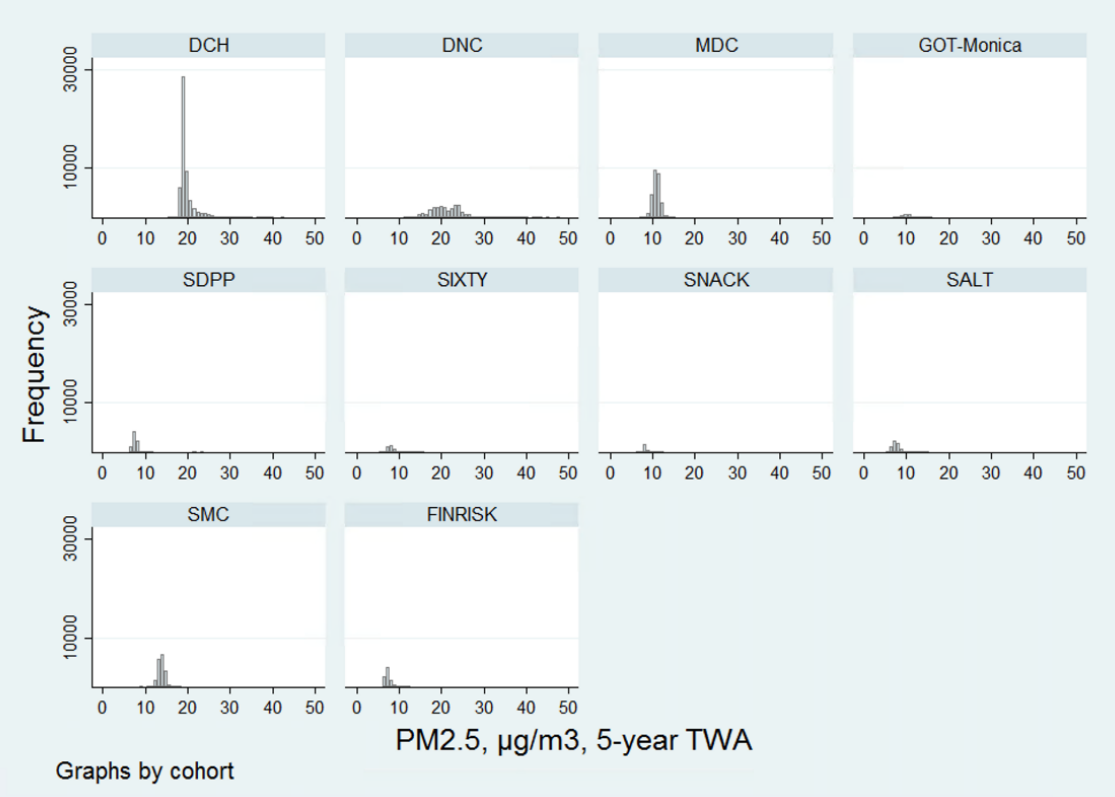

Figure S7: Cohort-specific histograms of exposure to NO_2.

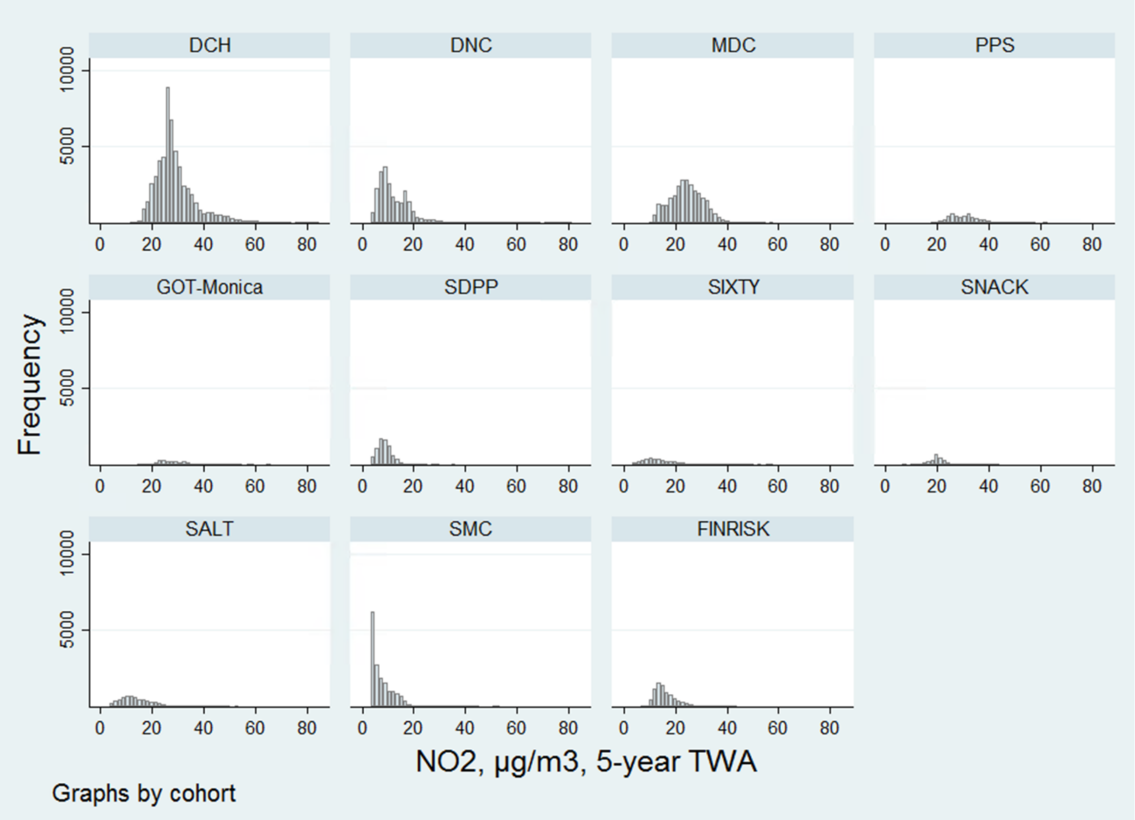

Figure S8: Overweight in relation to road traffic and railway noise exposure 5 years prior to baseline (OR and 95% CI per 10 dB L_{den}) according to different characteristics of the study subjects.

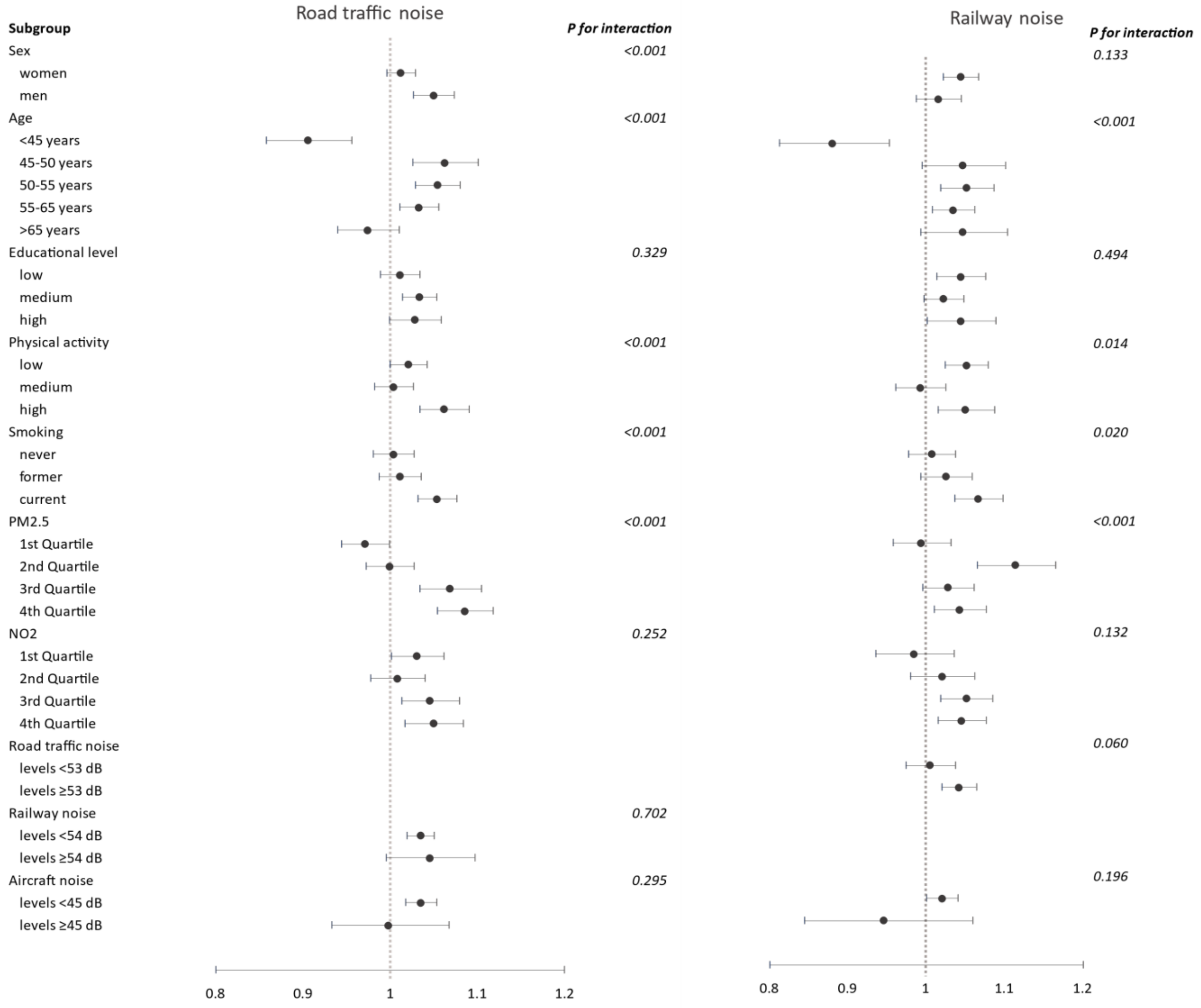

Note: Adjusted for cohort, sex, age, recruitment year (<1995, 1995-2000, >2000), educational level, marital status, area income, smoking status, and physical activity. Analytical samples differ for analysis with different interactors for road traffic and railway noise. Overweight: Body Mass Index ≥25 kg/m^2.

Figure S9: Central obesity in relation to road traffic and railway noise exposure 5 years prior to baseline (OR and 95% CI per 10 dB L_{den}) according to different characteristics of the study subjects.

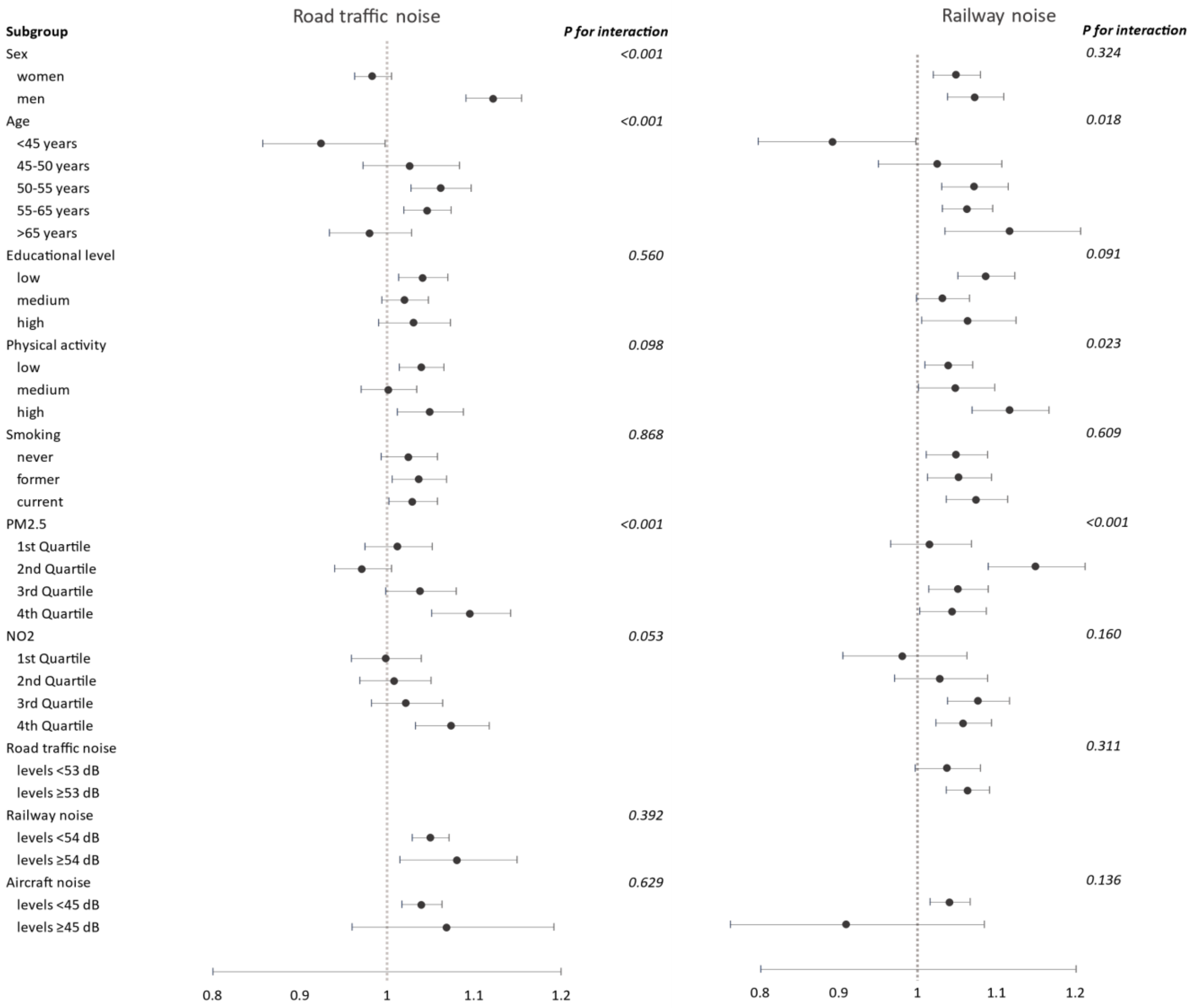

Note: Adjusted for cohort, sex, age, recruitment year (<1995, 1995-2000, >2000), educational level, marital status, area income, smoking status, and physical activity. Analytical samples differ for analysis with different interactors for road traffic and railway noise. Central obesity: Waist circumference in women: ≥88 cm, and in men: ≥102 cm.

Figure S10: Exposure-response associations between road traffic noise and overweight, obesity and central obesity, respectively, based on cohorts with measured outcomes only and additionally excluding the DCH in the lower panels.

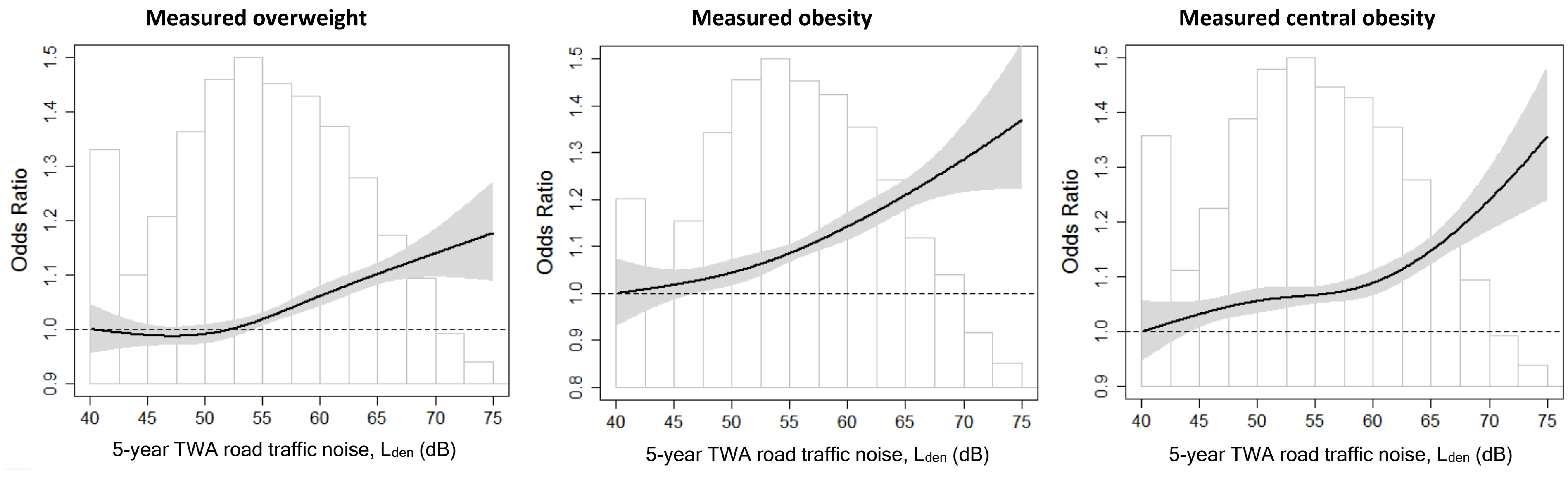

N=112,599; n=61,829 cases of overweight (including obesity)

N=66,572; n=15,896 cases of obesity.

N=104,833; n=25,144 cases of central obesity.

Measured overweight, excluding the DCH

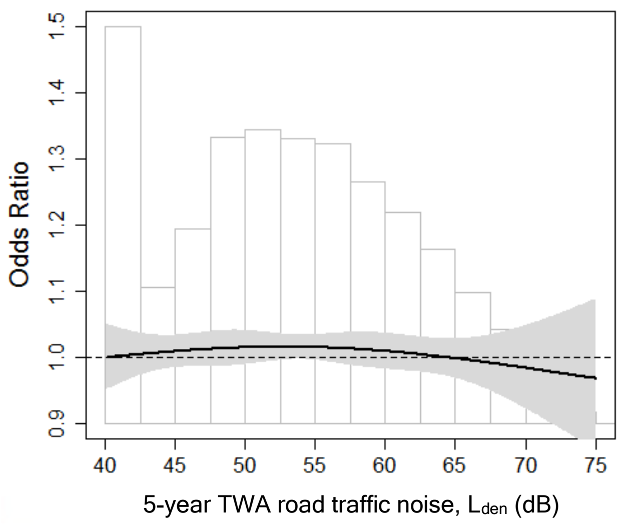

N=57,780; n=31,197 cases of overweight (including obesity)

Measured obesity, excluding the DCH

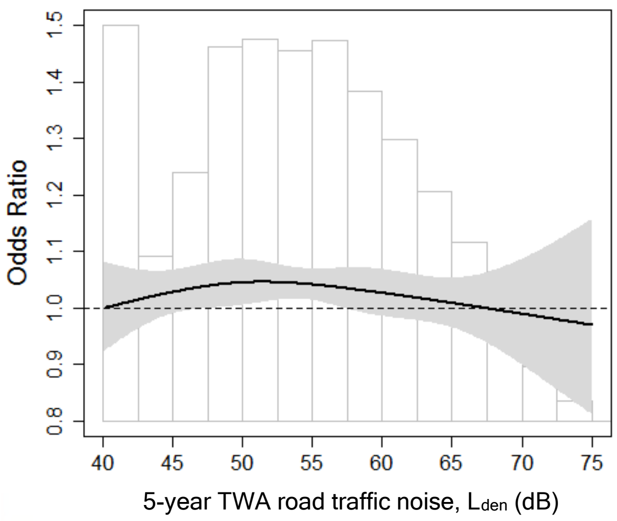

N=34,523, n=7,957 cases of obesity.

Measured central obesity, excluding the DCH

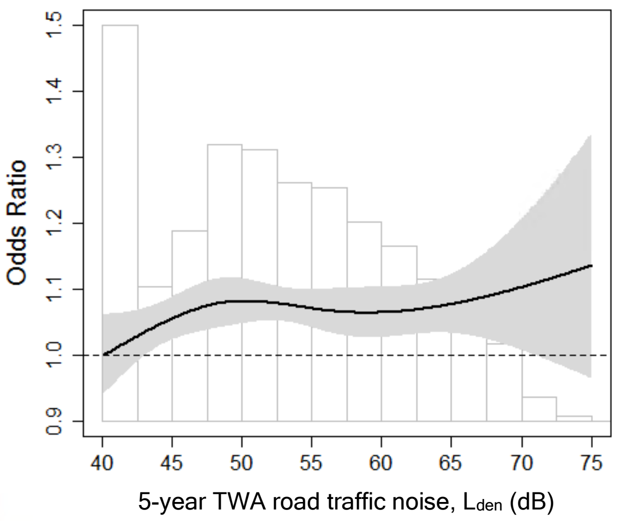

N=50,136, n=10,759 cases of central obesity.

Note: Natural splines with forced placement of knots at the 20th, 40th, 60th and 80th percentile. Adjusted for cohort, sex, age, recruitment year, educational level, marital status, area income, smoking status and physical activity.

TWA, Time-Weighted Average. Overweight: Body Mass Index ≥25 kg/m² compared to Body Mass Index <25 kg/m². Obesity: Body Mass Index ≥30 kg/m² compared to Body Mass Index <25 kg/m². Central obesity: Women: ≥88 cm compared to <88 cm; men: ≥102 cm compared to <102 cm.

Figure S11: Cohort-specific results performed through interaction analyses for overweight, obesity and central obesity in relation to road traffic noise (OR and 95% CI per 10 dB L_{den} increase).

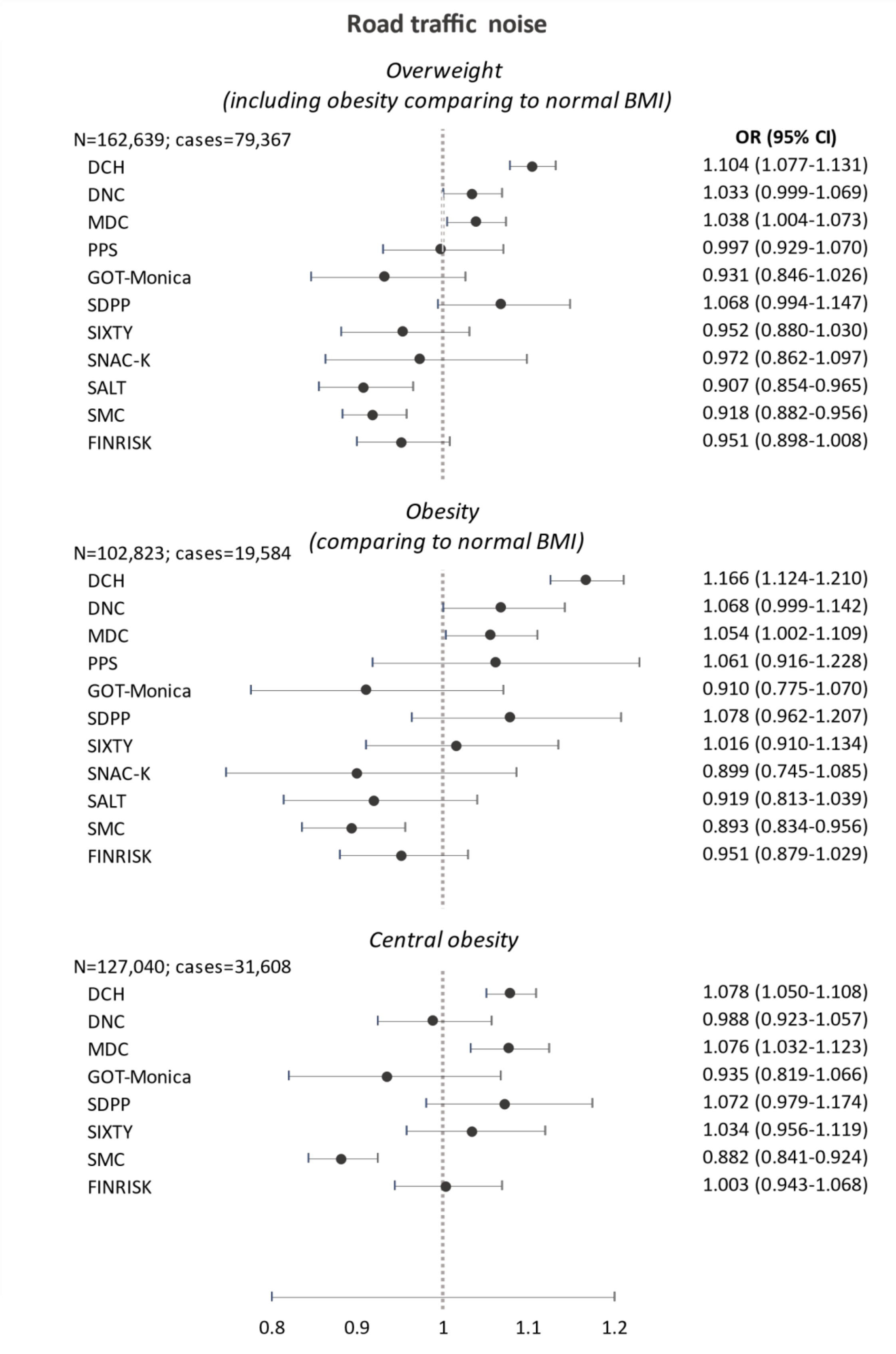

Note: DCH, Diet, Cancer and Health cohort; DNC, Danish Nurses Cohort; GOT-MONICA, Multinational Monitoring of Trends and Determinants in Cardiovascular Disease cohort (Gothenburg); MDC, Malmö Diet and Cancer Study; PPS, Primary Prevention Study cohort; SALT, Stockholm Screening Across the Lifespan Twin Study; SDPP, Stockholm Diabetes Prevention Program; Sixty, the Stockholm Cohort of 60-year-olds; SMC, The Swedish Mammography Cohort; SNAC-K, Swedish National Study of Aging and Care in Kungsholmen.
Adjusted for cohort, sex, age, recruitment year, educational level, marital status, area income, smoking status and physical activity.
Overweight: Body Mass Index ≥25 kg/m².
Obesity: Body Mass Index ≥30 kg/m².
Central obesity: Women: ≥88 cm, men: ≥102 cm.

Figure S12: Cohort-specific results performed through interaction analyses for overweight, obesity and central obesity in relation to railway noise (OR and 95% CI per 10 dB L_{den} increase).

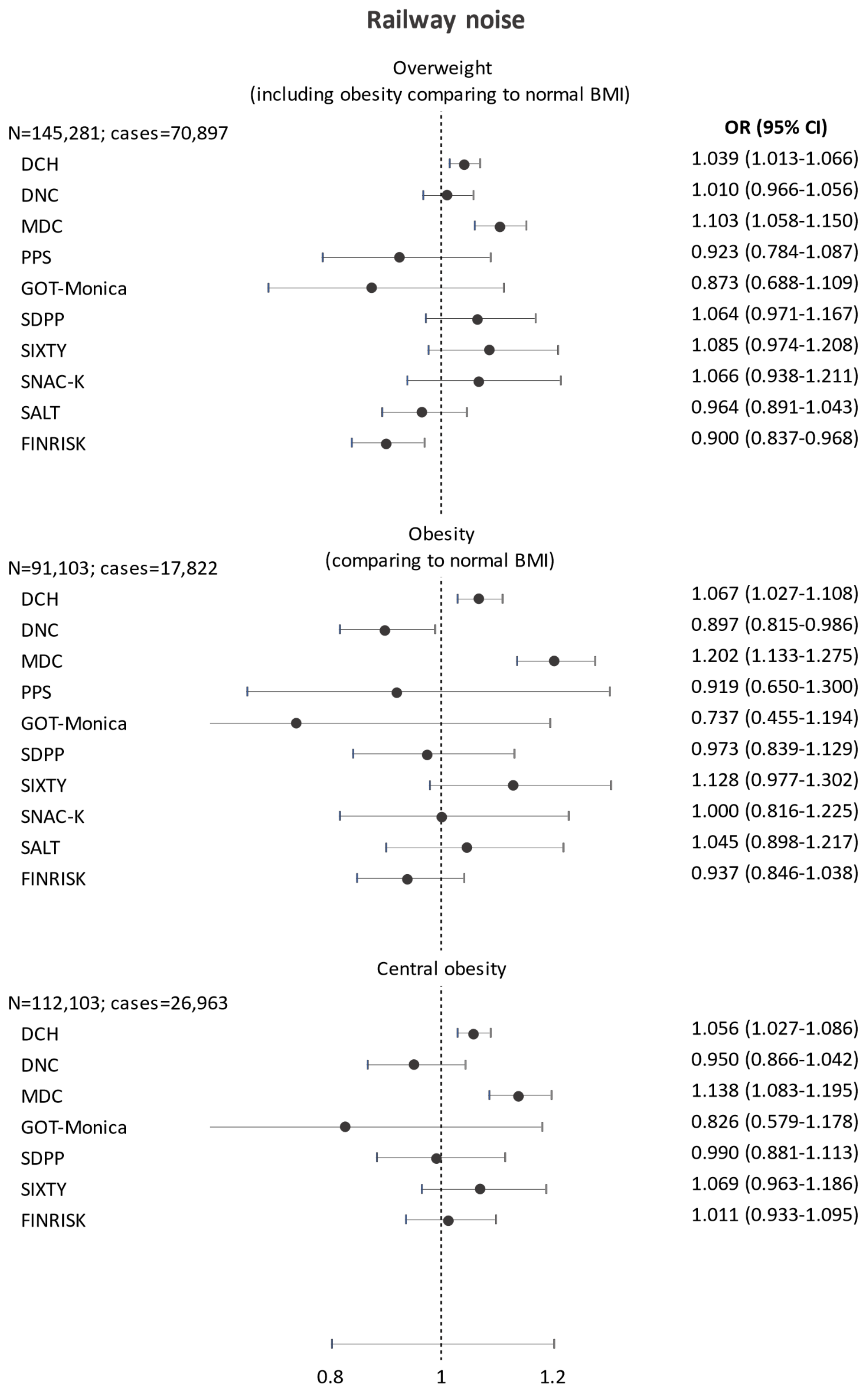

Note: DCH, Diet, Cancer and Health cohort; DNC, Danish Nurses Cohort; GOT-MONICA, Multinational Monitoring of Trends and Determinants in Cardiovascular Disease cohort (Gothenburg); MDC, Malmö Diet and Cancer Study; PPS, Primary Prevention Study cohort; SALT, Stockholm Screening Across the Lifespan Twin Study; SDPP, Stockholm Diabetes Prevention Program; Sixty, the Stockholm Cohort of 60-year-olds; SNAC-K, Swedish National Study of Aging and Care in Kungsholmen.
Adjusted for cohort, sex, age, recruitment year, educational level, marital status, area income, smoking status and physical activity.
Overweight: Body Mass Index ≥25 kg/m².
Obesity: Body Mass Index ≥30 kg/m².
Central obesity: Women: ≥88 cm, men: ≥102 cm.
